# Microtubule polarity determines the lineage of embryonic neural precursor in zebrafish spinal cord

**DOI:** 10.1038/s42003-024-06018-7

**Published:** 2024-04-10

**Authors:** Clément-Alexis Richard, Carole Seum, Marcos Gonzalez-Gaitan

**Affiliations:** Department of Biochemistry, Faculty of Science, University of Geneva, 30 Quai Ernest Ansermet, Geneva, 1205 Switzerland

**Keywords:** Mitotic spindle, Motor protein tracks, Developmental neurogenesis

## Abstract

The phenomenal diversity of neuronal types in the central nervous system is achieved in part by the asymmetric division of neural precursors. In zebrafish neural precursors, asymmetric dispatch of Sara endosomes (with its Notch signaling cargo) functions as fate determinant which mediates asymmetric division. Here, we found two distinct pools of neural precursors based on Sara endosome inheritance and spindle-microtubule enrichment. Symmetric or asymmetric levels of spindle-microtubules drive differently Sara endosomes inheritance and predict neural precursor lineage. We uncover that CAMSAP2a/CAMSAP3a and KIF16Ba govern microtubule asymmetry and endosome motility, unveiling the heterogeneity of neural precursors. Using a plethora of physical and cell biological assays, we determined the physical parameters and molecular mechanisms behind microtubule asymmetries and biased endosome motility. Evolutionarily, the values of those parameters explain why all sensory organ precursor cells are asymmetric in flies while, in zebrafish spinal cord, two populations of neural precursors (symmetric vs asymmetric) are possible.

## Abstract (web summary)

In Zebrafish embryos spinal cords, neural precursors are clustered in two distinct populations based on their spindle-microtubule enrichment. KIF16Ba, CAMSAP2a/3a and spindle-microtubule enrichment control Sara endosome motility and mediate neural precursor fates.

## Introduction

Asymmetric cell division (ACD) generates daughter cells with distinct cell fates, giving raise to cell diversity during the development of tissues. During ACD, specific molecules, so-called cell fate determinants, are unequally distributed between daughter cells. We have previously shown that Sara endosomes in dividing neural precursor (NP) cells of the zebrafish spinal cord could be asymmetrically segregated to one of the daughter cells during anaphase^1^. When NPs produce asymmetric lineages (so called *n•p* lineages), a daughter divides (*p* fate) and the sibling differentiates into a neuron (*n* fate). Asymmetric *n•p* lineages are generated by asymmetric cell division of the NP, which targets Sara endosomes and their Notch signaling cargo to one of the daughters, the one which will acquire the *p* fate.

In flies, asymmetric segregation of Sara endosomes during sensory organ precursor (SOP) mitosis, relies on two key features of the system: (i) the asymmetric density of microtubules (MTs) composing the central spindle, and (ii) the directed motility of Sara endosomes towards the plus end of MTs^2^. Importantly, this asymmetric Sara endosome targeting forecasts the fate of the SOP lineage. In vertebrates, however, the mechanism leading to the asymmetric segregation of Sara endosomes remains unknown. Furthermore, unlike flies, our previous study of Sara endosome asymmetric segregation in NP could not forecast whether its subsequent lineage is symmetric or asymmetric^1^.

Prompted by these issues, here we investigate (i) whether the composition of MTs at the central spindle underlies asymmetric motility of endosomes in zebrafish NP mitosis, (ii) whether the dynamic of MTs is predictive of the different types of NP lineages and (iii) what are the physical and cell biological basis of the mechanism mediating Sara segregation.

## Results

### Central spindle microtubules define two distinct types of neural precursors

**Fig. 1:**
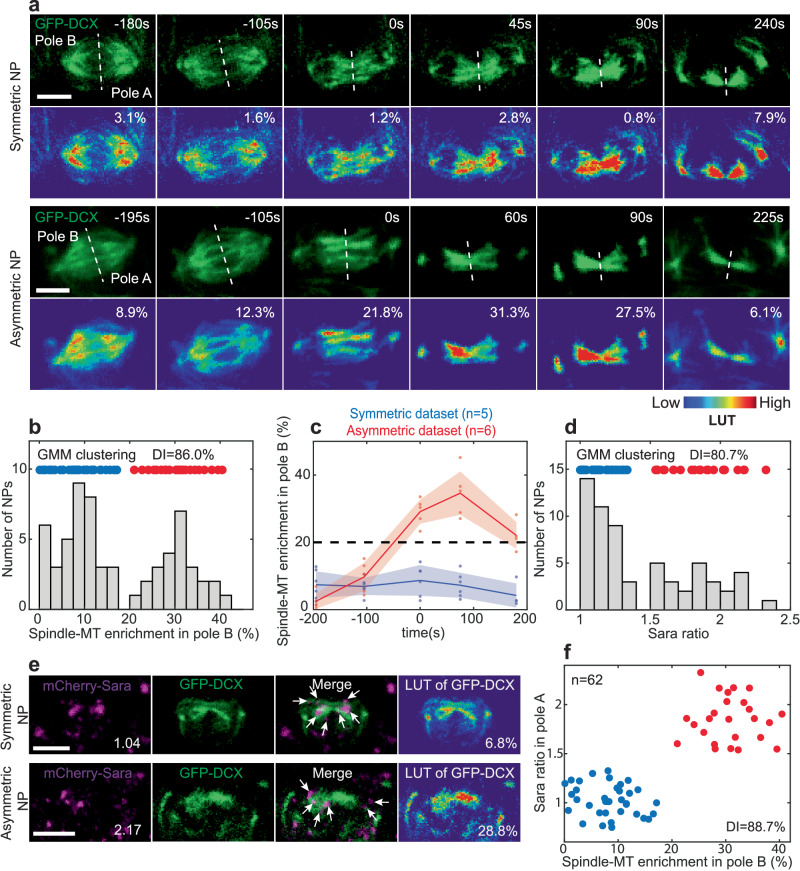
Two types of neural precursors: symmetric and asymmetric. **a** Sum z-projection time lapse of a symmetric and an asymmetric NP. Spindle-MTs are labeled with GFP-DCX (green). Look up table (LUT) shows spindle-MT density. White dashed line, midzone. Pole B (to the left) is defined as the pole with more spindle-MTs. Relative pole B percentage of enrichment and registered time are indicated. **b** Histogram of spindle-MT enrichment measured in pole B (*n* = 62 NPs). Above, data are clustered (GMM clustering analysis) into two groups (DI = 86.0%), symmetric (blue dots) and asymmetric (red dots) NPs. **c** Pole B mean dynamics of spindle-MT enrichment at the central spindle as a function of registered time for symmetric (blue, *n* = 5 NPs) or asymmetric (red, *n* = 6 NPs) NPs with respective individual data points (dots). Shade, standard deviation (SD). Dashed line, threshold for asymmetry according to the clustering analysis. **d** Histogram of Sara endosome ratio measured in pole having more Sara endosomes (*n* = 62 NPs). Above, symmetric/asymmetric clustered data (DI = 80.7%). **e** Sum z-projection of symmetric and asymmetric NPs (registered *t* = 75 s) showing spindle-MTs (GFP-DCX, green) and Sara endosomes (mCherry-Sara, magenta). Sara endosome ratio (in pole A) and percentage of spindle-MT enrichment (in pole B) are indicated. Arrows, Sara endosomes. **a**, **e** Scale bars, 5 μm. **f** Gaussian mixture model clustering (GMM) of Sara endosome ratio (in pole A) and spindle-MT enrichment (in pole B) (*n* = 62 NPs). Two clusters are found for symmetric (blue dots) and asymmetric NPs (red dots) with a high DI = 88.7%.

To understand the mechanism behind asymmetric targeting of Sara endosomes, we first studied the MTs of the central spindle during anaphase. We focused on somite 6 to 8 in embryos around 24 hours post-fertilization (hpf) (27 ± 1 somite stage). We monitored the density of central spindle MTs by following a tagged version of human Double Cortin (DCX)^3^ 75 ± 30 s after the onset of cytokinesis, which we define as the moment of appearance of the cleavage furrow (see methods) (Fig. 1a). In particular, we studied whether there is an asymmetry of MT density between the two sides of the central spindle which project into the two poles of the NP that will give rise to the two daughter cells.

We discovered that the distribution of MT asymmetry 75 s after the onset of cytokinesis is bimodal in the spinal cord (Fig. 1a, b). To validate the existence of two types of NPs according to microtubule density asymmetry, we performed clustering analysis. To determine whether there is more than one pool in the population of NPs, we used the Akaike Information Criterion (AIC), a test comparing the precision of models having different numbers of clusters. We studied this with two different clustering algorithms: Gaussian mixture model (GMM) using probability of distribution^4^ and K-mean model using centroid distances^5^ (colored dots in the x-axis of Fig. 1b; see also methods and Fig. S1a–d). We used those two independent clustering methodologies to reinforce our analysis. Both algorithms yield the same number of clusters and cluster composition (Fig. S1a–d). Then, we used the Dunn-index^6^ (DI, from zero to one), a metric where high values indicate reliable clustering, to evaluate the goodness of clustering of each population.

Figure 1b (bottom) shows that NPs are clustered into two distinct populations (high Dunn-index of DI = 86.0%): one cluster with low levels of asymmetry (so-called “symmetric”) and one with higher level of asymmetry. The asymmetric cluster represents 41% of the NPs and shows an average enrichment by 30 ± 5% in MT density in one of the poles. The two clusters are separated by a threshold of 20% enrichment, which we use hereafter as a criterium to separate the two NP pools. We defined pole A as the pole with lower spindle-MT density and pole B, that with higher spindle-MT density. In the asymmetric pool of NPs, enrichment raises during anaphase, peaks 75 s after cytokinesis onset and decays after that (Fig. 1c).

### Sara endosomes are enriched asymmetrically in the NP pool with asymmetric central spindle

We have previously shown that also Sara endosomes can be dispatched asymmetrically during NP mitosis^1^. We then wondered whether Sara asymmetry also defines two clusters of NPs and, if so, whether this correlates with the two pools of NPs according to their MT asymmetry. Figure 1d shows that, 75 s after cytokinesis onset, Sara endosomes ratio between the two daughters also show a bimodal distribution in the spinal cord, with two clusters separated by a threshold of 1.5-fold enrichment (DI = 80.7%). In the pool of asymmetric NPs, Sara endosomes are enriched by 1.8 ± 0.2 fold in one of the daughters.

We then evaluated MT and Sara endosome enrichments simultaneously in the same NPs. There is a tight correlation between the pool of NPs with MT asymmetry and Sara endosome asymmetry: if and only if an NP shows MT asymmetry above 20%, Sara is also enriched above 1.5-fold in the daughter with lower MT density (pole A; Fig. 1e, f). Cluster analysis combining these two traits (MTs and Sara), also uncovers two pools which are reliably separated (DI = 88.7%; Fig. 1f). The two pools correlate with the asymmetry of cortical Par3, a component of the Par complex which controls cell polarity and has previously been shown to be involved in asymmetric divisions of zebrafish NPs^7^. Indeed, Par3 enrichment in one daughter also defines two reliable pools (DI = 80.9%), which themselves correlate perfectly with MT asymmetry (Fig. S1e, f). However, as previously shown^1^, Par3 is not essential for Sara endosomes asymmetry (Fig. S1g). Consistently, we observed that both the symmetric and asymmetric pool of NPs, considering the distribution of MT densities, are present in Par3 morphants (DI = 92.0%; Fig. S1h).

### The asymmetric NP pool gives rise to asymmetric lineages

What are the fates of these two types of NPs? It is well established that, in the spinal cord, NPs divide a maximum of two times to produce three types of lineages: *n•p*, *n•n* and *p•p* which produce 3, 2 and 4 neurons, respectively^1,7–9^. In *n•p* lineages, the mother NP divides asymmetrically to produce a daughter that differentiates readily into a neuron (*n* daughter) and a daughter which is a progenitor cell (*p* daughter) that divides again to produce two neuron daughters itself. In *n•n* and *p•p* lineages, the mother cell divides symmetrically to produce either two neurons (*n•n*) or two progenitor cells that will give a lineage with four neurons (*p•p*).

**Fig. 2:**
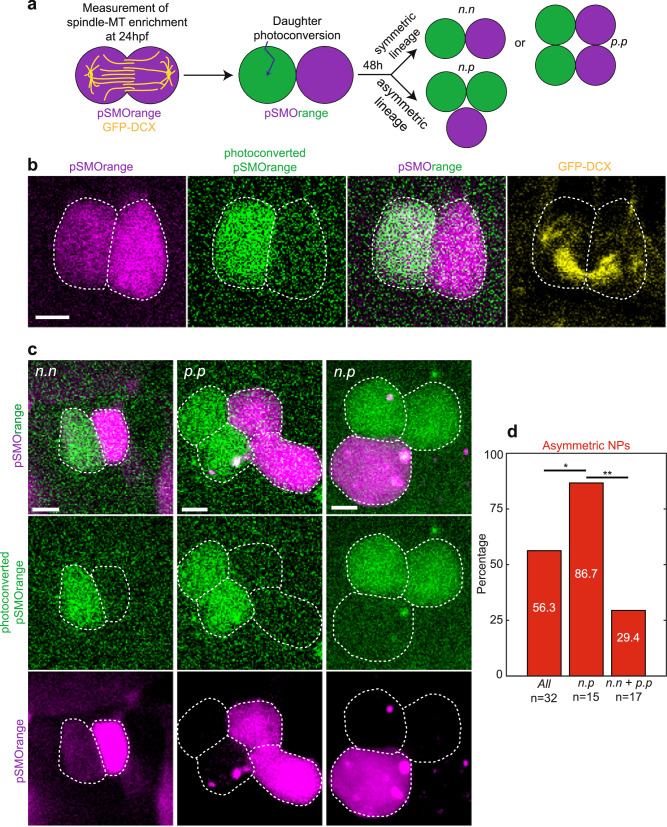
The asymmetric NP pool gives rise to asymmetric lineages. **a** Scheme of NP daughter photoconversion assay. After measurement of spindle-MT enrichment (GFP-DCX, yellow) in a single dividing NP (24hpf), pSMOrange was photoconverted (from red to far-red; green color). After 48 h, the composition of the lineage and the type of division pattern (*n•n*, *p•p* and *n•p*) of the photoconverted cell (here green) and the non-photoconverted cell (here magenta) were determined. **b** Maximal z-projection of a dividing 24hpf NP showing spindle-MTs (GFP-DCX, yellow) and pSMOrange (magenta /green; green is photoconverted). **c** Three maximal z-projections of the different lineages found 48 h after photoconversion. Lineages are assessed according to the resulting number of cells: 2 cells for *n•n*, 2 + 1 cells for *n•p*, and 2 + 2 cells for *p•p*. This assay allows to quantify the spindle-MT asymmetry in the mother NP and to trace independently the lineage of its two initial daughter cells after photoconversion. **b**, **c** Scale bars, 5 μm. **d** Percentage of asymmetric NPs (according to spindle-MT enrichment) for *all* photoconverted NPs (*n* = 32), NP which generated asymmetric lineages (*n•p*, *n* = 15) or symmetric lineages (*n•n* + *p•p*, *n* = 17), respectively. Chi-square test indicates that the asymmetry of MTs in the mother NP is statistically predictive of the type of subsequent lineage. 95% confidence; *p < 0.05 and **p < 0.01. Non indicated comparison, non-significant.

Lineage tracing was carried out by single-cell injection of mRNA encoding the photoconvertible protein pSMOrange^10^ in 32-cell-stage transgenic embryos expressing GFP-DCX. We first imaged MTs at 27 ± 1 somite stage in the 6–8 somite region by following GFP-DCX signal in a mother NP during anaphase and then photoconverted pSMOrange later in one of the daughters (Fig. 2a, b). Two days later, we determined the lineage from each daughter (Fig. 2c). *A posteriori*, to avoid bias, we analyzed the spindle-MT enrichment of the mother NP to determine whether the mother division was symmetric or asymmetric and, if asymmetric, whether the photoconverted daughter was the one enriched in MTs.

Figure 2d shows that most *n•p* lineages (13 out of 15 lineages, *p* = 4.88E-04, binomial test) are generated by asymmetric mother NP divisions. Conversely, symmetric lineages (*n•n* or *p•p*) are significantly biased to be generated by symmetric NPs (12 out of 17, *p* = 2.24E-02, binomial test; see also Fig. S1i). Furthermore, when the NP division is asymmetric, the daughter with a lower density of MTs (pole A), is almost always acquiring the precursor fate and divides again (92.3%; 12 out of 13 NPs, *p* = 1.22E-04, binomial test). Our observations indicate that there are two distinct, non-overlapping populations of NPs in the spinal cord (symmetric *vs* asymmetric according to MTs or Sara segregation) which give rise to different symmetric or asymmetric lineages; in the asymmetric lineage, biased inheritance of Sara endosomes in a daughter forecasts her fate as a progenitor daughter cell, which undergoes another round of mitosis before their two daughters differentiate into a neuron.

### Analysis of endosomal motility on spindle-microtubules

**Fig. 3:**
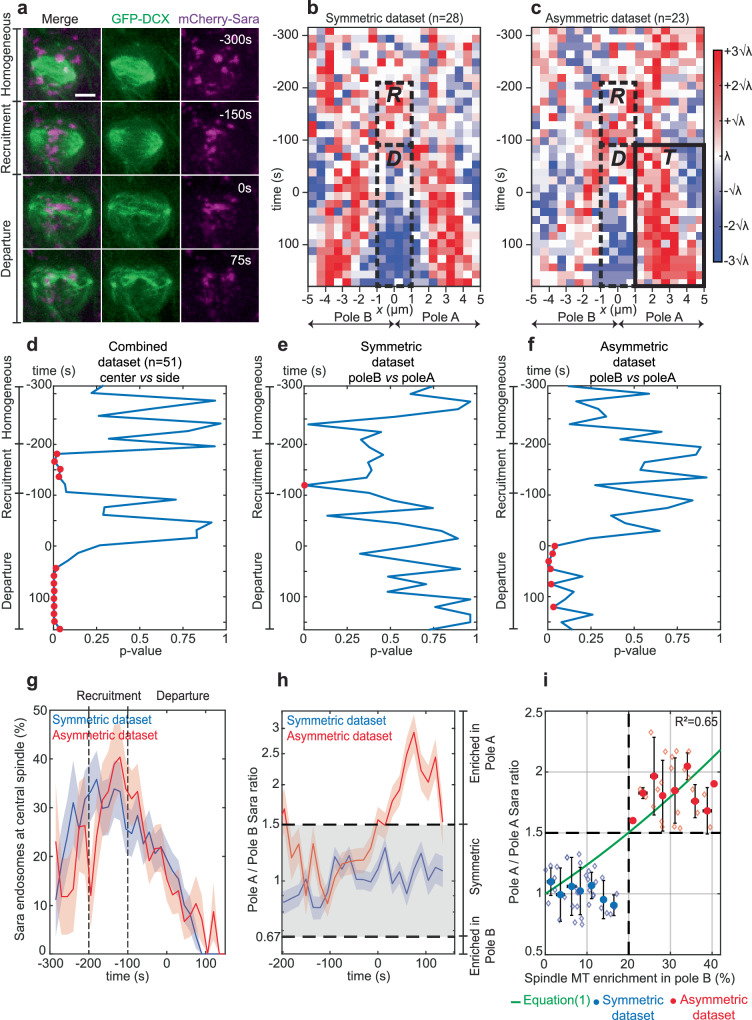
Analysis of endosomal motility on spindle-microtubules. **a** Maximal z-projection time lapse of a 24hpf dividing NP showing spindle-MTs (GFP-DCX, green) and Sara endosomes (mCherry-Sara, magenta). Sara endosomes are initially homogeneously distributed (*t* = −300 s), then they are recruited to central spindle (*t* = −150 s) before their progressive departure and segregation in the forming daughter cells (*t* = 0 s and *t* = 75 s). Scale bar, 5 μm. t corresponds to register time. Sara endosomes densities in space and time for symmetric NPs (**b**; *n* = 28 NPs and 4503 endosomes) and asymmetric NPs (**c**, *n* = 23 NPs and 2994 endosomes). LUT indicates high (red), or low (blue) Sara endosomes densities as compared to random Poisson distributions using the λ(t) of Poisson. For each registered time interval, the value of λ(t) is calculated from the mean number of endosome per bin (see methods). Boxes indicate the regions and intervals of time in which Sara endosomes are recruited to the central spindle (*R*, −200 s to −100 s), depart from the central spindle (*D*, −100 s to 150 s) or are targeted asymmetrically to pole A (*T*, −100 s to 150 s) in asymmetric NPs (**c**). ANOVA comparison of Sara endosomes mean densities as a function of registered time between cell center and cell sides (**d**) or pole B and pole A (**e**, **f**) for combined (**d**; *n* = 51 NPs, 7497 endosomes), symmetric (**e**) and asymmetric datasets (**f**). Cell center: *x* from −1 μm to 1 μm; cell sides (pole A/pole B) as in (**b**), excluding from −1 µm to 1 µm. Red dots indicate a statistically significant difference of density (*p* < 0.05) and confirm the recruitment and departure phases and the asymmetric segregation of Sara endosomes towards pole A in the asymmetric, but not in the symmetric NPs. **g** Average percentage of Sara endosomes in the central spindle area as a function of registered time for the symmetric (blue) and asymmetric (red) datasets. In both datasets, homogeneously located endosomes are targeted toward central spindle during recruitment before their progressive departure (black dashed lines). **h** Dynamic of Sara endosomes mean ratio (log scale) as a function of registered time in pole A for symmetric (blue) and asymmetric (red) datasets. Gray area indicates symmetric ratio of endosome between pole A and pole B. Ratio above 1.5-fold indicate an asymmetry in pole A and ratio below 0.67-fold (1/1.5) indicate an asymmetry in pole B. **g**, **h** Shades, relative standard error mean (RSEM). **i** Sara endosome ratio in pole A as a function of spindle-MT enrichment in pole B. Symmetric (blue) and asymmetric (red) NPs are binned from individual data points (diamonds). Green, Eq. 1. R², correlation coefficient of Eq. 1 with experimental data (95% confidence). Black dashed lines indicate thresholds of separation for symmetric/asymmetric NPs according to the clustering analysis (Fig. 1b, d).

Because the symmetric/asymmetric motility of Sara endosomes forecasts the type of lineages and the fate of daughter cells upon NP division, we then wondered what controls the motility of this organelle. We therefore first analyzed the dynamics of endosomal targeting in these two NP populations by following mCherry-Sara and GFP-DCX (Fig. 3a). We automatically tracked Sara endosomes in dividing NPs using Trackmate^11^ together with a custom Matlab code (for details, see Fig. S2a, b and methods). Sara endosome tracks were registered in time with respect to the onset of cytokinesis (*t* = 0 s). Onset of cytokinesis was detected by a characteristic tilting of the two poles of the dividing cell which happens at the same time as the formation of the cytokinetic cleavage furrow (Fig. S2b–f). Spatial registration of the tracks was based on the position of the centrosomes and the midpoint between those, which corresponds to *x* = 0 µm (Fig. S2a, b).

Figure 3b, c show spatiotemporal density plots considering Poisson statistics (see methods and Fig. S3a) for the tracks of NPs with symmetric and asymmetric Sara endosomes, as well as a randomized data set as a control (Fig. S3b–f). We first studied the targeting of endosomes towards the central spindle region, defined as a 2 µm wide region around the midpoint (dashed boxes in Fig. 3b, c; Fig. S2a). This corresponds to the antiparallel array of MTs of the central spindle, as monitored by localization of mCherry-MKLP1^12^ (Fig. S3g). We analyzed central spindle targeting by ANOVA statistics (Fig. 3d–f and Fig. S3h, i), where a *P*-value below 0.05 (red dots) in a particular time interval indicates that the density of endosomes has a statistically significant heterogeneity, i.e., it is increased or decreased in a particular region compared to the rest of the cell. Endosomes are indeed recruited to the central spindle between −200 s and −100 s (Fig. 3a, d, g). After that, endosomes depart from the central spindle (Fig. 3a, d, g). Similar ANOVA analysis comparing the enrichment between the two poles shows that, in asymmetric NPs, endosomes are enriched in pole A (with less MTs) from *t* = 0 s to *t* = 150 s (Fig. 3c, f). In contrast, no asymmetry between the two poles can be detected in symmetric NPs after departure (Fig. 3b, e).

Like in the case of MTs, in the NP pool with asymmetric Sara endosomes, asymmetry raises during anaphase, peaks 75 s after cytokinesis onset and decays after that (Fig. 3h). It is worth noting that, because of this decay, if the ratio of Sara endosomes is evaluated after 75 s, the two clusters merge (Fig. S4a, b), consistent with our own previous report^1^. This also explains that if evaluated later, the asymmetry of Sara endosomes could not forecast whether the subsequent lineage was symmetric or asymmetric^1^.

We have previously shown^2^, based on a theoretical model of plus-end directed endosomal motility on an antiparallel, asymmetric MT overlap (like in the central spindle), that the steady-state endosome distribution is captured by the expression:

1 $$\frac{{{{{{{\rm{P}}}}}}}^{{{{{{\rm{A}}}}}}}}{{{{{{{\rm{P}}}}}}}^{{{{{{\rm{B}}}}}}}}=\frac{1+\Delta }{1-\Delta }\exp \left(\frac{2{{{{{{\rm{k}}}}}}}_{{on}}{{{{{\rm{\rho }}}}}}{{{{{\rm{vl}}}}}}\Delta }{{{{{{\rm{D}}}}}}{{{{{{\rm{k}}}}}}}_{{off}}}\right)$$

with ${P}^{{{\mbox{A}}}}$, ${P}^{{{\mbox{B}}}}$, the probabilities for an endosome to be in either side of the antiparallel overlap; $\rho =({\rho }_{A}+{\rho }_{B})/2$ and $\varDelta =({\rho }_{A}-{\rho }_{B})/({\rho }_{A}+{\rho }_{B})$ with ρ_a_, ρ_b_, MT densities in pole A and pole B, respectively; ${{{\mbox{k}}}}_{{{\mbox{on}}}}$,${{{\mbox{k}}}}_{{{\mbox{off}}}}$, MT association/dissociation constants of the motor; $v$, the endosome motor-driven velocity; ${{\mbox{D}}}$, the diffusion coefficient of endosomes detached from MTs and ${{\mbox{l}}}$, the antiparallel overlap length.

Based on Eq. 1, Fig. 3i shows how the fraction of endosomes in pole A depends on the enrichment of MTs in pole B. To evaluate the expected endosomal asymmetry, we previously measured the values of all the key dynamic parameters in this system (see methods, Figs. S3g; S4c–f). Thus, we studied the mean square displacement (MSD) of endosome tracks to measure the diffusion coefficient (D). The directed velocity of the endosomes ($v$), the rates of unbinding (${k}_{{off}}$) and binding (${k}_{{on}}\rho$) of endosomes to MTs were measured from the duration of the episode in which endosomal movement is directed or diffusive, respectively, and the length of the antiparallel array of MTs (l) by using mCherry-mKLP1 labeling. We estimated *D* = (8.15 ± 0.46)10^−3^ μm^2^ s^−1^ (Fig. S4c, d), *v* = (1.36 ± 1.07)10^−1 ^µm *s*^−1^, *l* = (1.3 ± 0.1)µm (Fig. S3g), ${k}_{{off}}=(0.97\pm 0.33){s}^{-1}$ (Fig. S4e) and ${k}_{{on}}\rho =(0.057\pm 0.004){s}^{-1}$ (Fig. S4f).

Plugging these experimental parameters in Eq. 1, a 30% MT enrichment in pole B causes a 1.8-fold enrichment of Sara endosomes in pole A (Fig. 3i), which coincides precisely with the values of bias observed experimentally in an independent experiment (*cf*. Fig. 1b, d). This suggests that plus-end endosomal motility on an antiparallel, asymmetric MT overlap can explain in quantitative terms the asymmetry of endosomes observed in NPs of the spinal cord of zebrafish.

Comparing the dynamics of Sara endosomes targeting in fly SOPs and zebrafish NPs, the time spent at central spindle (residence time) and the timing of departure differ significantly. Indeed, in NPs, the residence time is 2.5-fold shorter than in SOPs (~500 s in SOP vs. ~205 s in NP; Fig. 3b–d, g). Consequently, in NPs, the departure of Sara endosomes from the central spindle is almost completed when the cytokinetic cleavage furrow first appears (Fig. 3), while, in SOPs, Sara endosomes departure and segregation is completed significantly later^2^. In addition, considering the physical parameters of Sara endosomes motility in SOPs or NPs plugged in Eq. 1, we found that for a 30% spindle-MT enrichment the asymmetry of Sara endosomes is ~2.4-fold higher in SOP than in NP. This difference of the level of endosomal asymmetry is due to the diffusion coefficients: $D=\left(2.1\pm 0.1\right){10}^{-3}\mu {m}^{2}{s}^{-1}$ in SOPs^2^ which is ~4-fold smaller than $D=\left(8.15\pm 0.46\right){10}^{-3}\mu {m}^{2}{s}^{-1}$ in NPs. This lower levels of Sara endosomes asymmetry in NP compared to SOPs could arise from the requirement of different NPs in the spinal cord to generate both symmetric and asymmetric progenies. This control requires therefore more flexibility than in fly SOPs where endosomal segregation must be asymmetric for all the cells. Those differences for diffusion, residence times at the central spindle and symmetry of the spindle shed light on species-specific adaptations in molecular mechanisms and temporal dynamics.

### Kif16Ba is the motor of the Sara endosomes

**Fig. 4:**
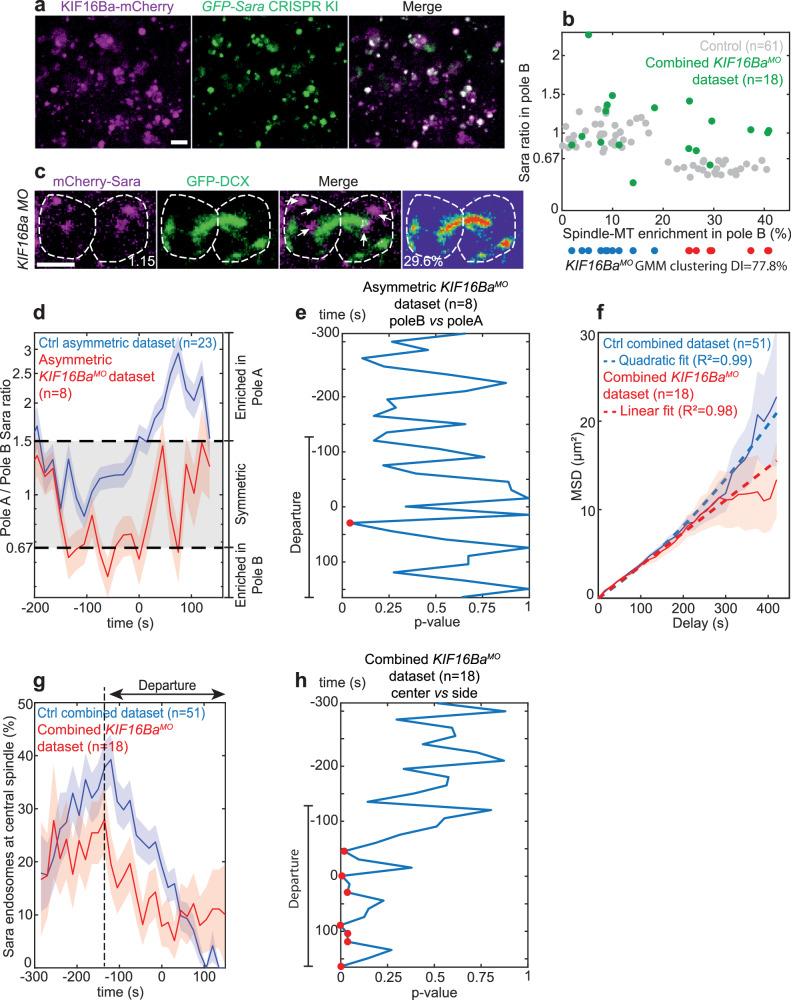
KIF16Ba is the motor of Sara endosomes. **a** Maximal z-projection of 24hpf zebrafish spinal cord between somites 6–8, showing KIF16Ba (KIF16Ba-mCherry, magenta) and Sara endosomes (GFP-Sara CRISPR Knock in, green) colocalization. **b** Relative pole B Sara endosomes ratio as a function of spindle-MT enrichment for control (gray, *n* = 61 NPs) and *KIF16Ba*^*MO*^ (green, *n* = 18 NPs) datasets. Below, GMM clustering of *KIF16Ba*^*MO*^ spindle-MT enrichment (DI = 77.8%) with symmetric (blue) and asymmetric (red) clusters. **c** Sum z-projection of a dividing *KIF16Ba*^*MO*^ asymmetric NP (registered *t* = 75 s) showing Sara endosomes (mCherry-Sara, magenta) and spindle-MTs (GFP-DCX, green). Relative pole B Sara endosomes ratio and spindle-MT enrichment are indicated. Arrows show Sara endosomes symmetric inheritance between the poles (cell contours). **a**, **c** Scale bars, 5 μm. **d** Dynamic of Sara endosomes mean ratio (log scale) as a function of registered time in pole A for control asymmetric (blue; *n* = 21 NPs) and *KIF16Ba*^*MO*^ asymmetric (red; *n* = 8 NPs) datasets. ANOVA comparison of Sara endosome mean densities as a function of registered time between pole B and pole A (**e**) or cell center and cell sides (**h**) for *KIF16Ba*^*MO*^ asymmetric (**e**; *n* = 8 NPs, 1141 endosomes) and *KIF16Ba*^*MO*^ combined datasets (**h**; *n* = 18 NPs, 1986 endosomes). **f** Weighted average mean square displacement (MSD) (weighted according to certainty, see methods) as a function of delay for control combined (blue line) and *KIF16Ba*^*MO*^ combined (red line) datasets. Blue dashed line, quadratic fit of combined control dataset. Red dashed line, linear fit of *KIF16Ba*^*MO*^ combined dataset. R², correlation coefficient (95% confidence). **g** Average percentage of Sara endosomes in the central spindle area as a function of registered time for the control combined (blue) and *KIF16Ba*^*MO*^ combined (red) datasets. Black dashed line indicates departure. **d**, **g**, **f** Shades, relative standard error mean (RSEM).

What motor drives the plus end motility of endosomes? In flies, Sara endosomes motility is mediated by the Klp98A kinesin^2^. In zebrafish, its homolog KIF16Ba, colocalizes with Sara endosomes (Fig. 4a). In *KIF16Ba* morphants, when considering MT asymmetry, two populations of NPs were observed: the frequency of asymmetric NPs is similar to that in wildtype animals (44.4%; with DI = 77.8% for the confidence of clustering; Fig. 4b). In contrast, no asymmetric Sara endosomes targeting was found in the population of NPs with asymmetric MTs (Fig. 4c–e). Injection of KIF16Ba mRNA in *KIF16Ba* morphant embryos rescued the asymmetric inheritance of Sara endosomes in asymmetric NPs (Fig. S5a). This is consistent with the idea that KIF16Ba is essential for the motility of Sara endosomes.

Indeed, MSD analysis shows that the movement of Sara endosomes in *KIF16Ba* morphant NPs is merely diffusive (confined diffusion; see Methods), without a directed component (Fig. 4f and Fig. S5b, c). As a consequence, Sara endosomes fail to be targeted first to the central spindle (Fig. 4g, h) and later, to be dispatched asymmetrically (Fig. 4b–e and Fig. S5d, e). Indeed, comparison of the recruitment phase between WT and *KIF16Ba* morphant uncovers a statistically significant difference of Sara endosomes percentage at central spindle (Fig. 4g; two-sample Kolmogorov–Smirnov test, *p* < 0.001). It is worth noting that, while endosomal enrichment in the central spindle is impaired in *KIF16Ba* morphants, endosomes which are located at the central spindle by chance, still depart from there like in wildtype (Fig. 4g, h and Fig. S5d, e). This suggests that the mechanism of departure involves phenomena other than those implicating KIF16Ba.

### Machinery behind the generation of asymmetric central spindle

**Fig. 5:**
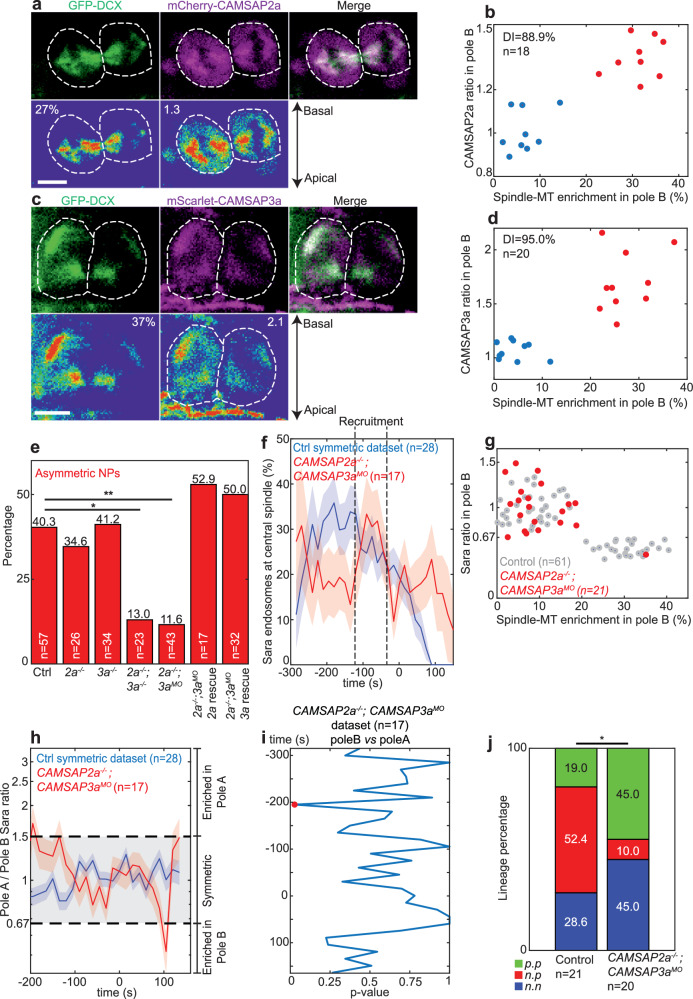
CAMSAPs and asymmetric spindle-MTs. Sum z-projection of a dividing NP showing spindle-MTs (GFP-DCX, green) and CAMSAP2a (mCherry-CAMSAP2a, magenta; **a**) or CAMSAP3a (mScarlet-CAMSAP3a, magenta; **c**). Below, LUT shows respective densities and relative pole B spindle-MT enrichments, and CAMSAP2a (**a**) or CAMSAP3a (**c**) ratio are indicated. Dashed lines, cell contours. Apico-basal axis is indicated. GMM clustering of relative pole B spindle-MT enrichment as a function of CAMSAP2a (**b**; *n* = 18 NPs, DI = 88.9%) or CAMSAP3a (**d**; *n* = 20 NPs, DI = 95.0%) ratio. Two clusters are found for symmetric (blue) and asymmetric (red) NPs. CAMSAP2a and CAMSAP3a ratio positively correlate with spindle-MT enrichment. **e** Percentage of asymmetric NPs in control and various *CAMSAP2a* and *CAMSAP3a* mutant, morphant and rescue combinations. **f** Average percentage of Sara endosomes in the central spindle region as a function of registered time for the control symmetric dataset (blue; *n* = 28 NPs and 4503 endosomes) and *CAMSAP2a*^*-/-*^*; CAMSAP3a*^*MO*^ dataset (red; *n* = 17 NPs and 1430 endosomes). Black dashed lines indicate recruitment and departure. **g** Relative pole B Sara endosomes ratio as a function of spindle-MT enrichment for control (gray, *n* = 61 NPs) and *CAMSAP2a*^*-/-*^*; CAMSAP3a*^*MO*^ (*n* = 21 NPs) datasets. **h** Dynamic of Sara endosomes mean ratio (log scale) as a function of registered time in pole A for symmetric (blue) and *CAMSAP2a*^*-/-*^*; CAMSAP3a*^*MO*^ (red) datasets. **f**, **h** Shades, relative standard error mean (RSEM). **i** ANOVA comparison of Sara endosomes mean densities as a function of registered time for pole B *vs* pole A in *CAMSAP2a*^*-/-*^*; CAMSAP3a*^*MO*^ dataset. **j** Percentage of *n•n* (blue), *n•p* (red), and *p•p* (green) lineages from mother NP photoconversions in *control* or *CAMSAP2a*^*-/-*^*; CAMSAP3a*^*MO*^. **e**, **j** Chi-square test, 95% confidence, *p < 0.05, **p < 0.01. Other comparisons are non-significant.

We then studied the machinery responsible to achieve an asymmetric central spindle in asymmetric NPs. It has previously been shown in flies that asymmetry of MTs in the central spindle is mediated by stabilization of the minus end of MTs by Patronin^2^, the fly ortholog of Calmodulin-Regulated Spectrin-Associated Proteins (CAMSAPs) in vertebrates^13^. Some CAMSAPs are MT associated proteins which bind minus ends^14^. In zebrafish, based on sequence homology, we found six CAMSAP proteins (Fig. S6a). Of these, CAMSAP2a and CAMSAP3a are found associated to central spindle MTs (Fig. 5a, c). In asymmetric NPs, CAMSAP2a is also enriched asymmetrically, and it is symmetric in symmetric NPs (Fig. 5a, b). CAMSAP3a is polarized, appears mainly apically and associates with MTs (Fig. 5c and Fig. S6b, c). During mitosis, CAMSAP3a is distributed asymmetrically in asymmetric NPs, similar to CAMSAP2a (Fig. 5d).

Single morphants for *CAMSAP2a* or *CAMSAP3a* did not show a phenotype in the number of NPs which show asymmetric microtubule density (Fig. S6d). Single CRISPR mutants for *CAMSAP2a* or *CAMSAP3a* (Fig. S6e, f) did not show asymmetric phenotype either (Fig. 5e). However, double *CAMSAP2a*^*-/-*^; *CAMSAP3a*^*-/-*^ mutants show a dramatic depletion of the pool of NPs with asymmetric MT density in the central spindle (Fig. 5e, see also Fig. S6g). A *CAMPSAP2a*^*-/-*^ mutant which is morphant in addition for *CAMSAP3a* (*CAMSAP2a*^*-/-*^*; CAMSAP3a*^*MO*^) shows the same MT phenotype as the double morphant or mutant and could be rescued by injection of mRNA for mCherry-CAMSAP2a or mScarlet-CAMSAP3a (Fig. 5e). Double mutants and morphants also show somitogenesis and segmentation defects (Fig. S6h–k) as observed in other studies where Notch/Delta signaling was impaired^15–17^. In *CAMSAP2a*^*-/-*^*; CAMSAP3a*^*MO*^, Sara endosomes are targeted to the central spindle like in control cells (Fig. 5f, Fig. S7a–c), but are never targeted asymmetrically (Fig. 5g–i) consistent with the depletion of the asymmetric pool of NPs (Fig. 5e).

Taken together, these data uncover a scenario where CAMSAP2a and CAMSAP3a are enriched asymmetrically in pole B in asymmetric NP cells, where they could stabilize spindle-MTs and locally enrich their density, consistent with previous reports^2,13,18^. This mediates asymmetric motility and targeting of Sara endosomes to pole A. As a consequence of the lack of NPs with spindle-MTs and Sara endosomes asymmetries, the frequency of *n•p* lineages is drastically reduced in *CAMSAP2a*^*-/-*^*; CAMSAP3a*^*MO*^ animals (Fig. 5j). This confirms in addition that the asymmetric NP neuroblasts generate *n•p* lineages (Fig. 2d).

## Discussion

The generation of a complex nervous system, such as that of vertebrates, is mediated by a plethora of mechanisms, of which ACD plays a prominent role. We found that in the spinal cord of zebrafish, diversity is generated by the existence of distinct pools of NPs with different fates, giving rise to symmetric or asymmetric lineages. Those NP pools are characterized by the symmetry or asymmetry of their central spindle during mitosis, which drive asymmetric targeting of Sara endosomes. Supporting this concept our key observations are: (i) NPs in the spinal cord can be clustered into two distinct pools where central spindle is asymmetric or not (Fig. 1a, b), (ii) central spindle asymmetry forecasts whether a lineage will be symmetric or asymmetric (Fig. 2d), (iii) the asymmetric dispatch of Sara endosomes is fully forecasted by the situation in the central spindle (Fig. 1e, f), (iv) while the asymmetry of central spindle and endosomes is forecasted by the symmetry/asymmetry of Par3 (Fig. S1e, f), Par3 is dispensable to generate those asymmetries^1^ (Fig. S1g, h), (v) the motility of endosomes in the central spindle is directed by KIF16Ba (Fig. 4g, h) and (vi) the asymmetry of the central spindle is mediated by asymmetry of CAMSAP proteins (Fig. 5a–e), (vii) asymmetric targeting of endosomes in an asymmetric central spindle depends on the binding and processivity of the kinesin, the diffusion coefficient of endosomes and the length of the region containing antiparallel MTs in the central spindle (Fig. 3i). We therefore established the existence of different pools of NPs with different fates, the origin of those pools and the physical and molecular mechanism of asymmetric dispatch of a signaling organelle, the Sara endosomes.

While we found that the existence of a distinct NP pool with central spindle asymmetry depends on the asymmetry of CAMSAP proteins, it is however unclear how CAMSAP distribution is controlled. An interesting candidate is Katanin: a negative regulator of MT-minus-end stabilization; it forms a complex with CAMSAPs and counteracts the formation of CAMSAP-decorated MT lattices^19,20^. Consistently, depletion of Katanin in zebrafish impairs the asymmetric cell division of NPs^21^. Therefore, it could be interesting to analyze if Katanin depletion plays a role in the asymmetry of spindle-MT enrichment and Sara endosomes segregation.

The apico-basal orientation of the NP division and its link to NP fate has previously been studied in a number of reports^22^. Horizontal or oblique divisions induce different segregation of apically polarized proteins such as Par3^7,23^. It is possible that other factors in the Par asymmetry complexes are asymmetrically segregated during oblique divisions playing a role in cell fate determination^24^.

We also uncover that, while Sara endosomes residence time at central spindle is different between SOPs and NPs, their departure from central spindle takes a similar time (~200 s of departure event in both organisms). In SOPs, we found that departure is mediated by Notch and its binding to Uninflatable and by phosphorylation of Sara itself ^25,26^. Since the shift between recruitment and departure dynamics happens earlier in NPs than SOPs, it indicates that the temporal activation of the mechanisms controlling Sara endosomes motility is different between zebrafish and flies.

In addition, we found that the plus end directed motor KIF16Ba is not necessary for the final departure of endosomes from the central spindle (Fig. 4g). This is not too surprising, because the final departure must implicate the movement of endosomes towards the minus end of MTs at the outer side of the central spindle. This points to Dyneins as candidates for this step, as recently proposed^23^. We however did not find a key role for Dyneins on this step of departure (Fig. S8a–c). Indeed, *Dlic1* morpholino injection did not prevent departure of Sara endosomes from the cleavage furrow during NP division.

Therefore, our results uncover that the physics of the asymmetric endosomal targeting mechanism, based on asymmetric MT cytoskeleton, is similar to those found in insects. This indicates that this mechanism had been conserved since their last common ancestor, the *Urbilaterian* more than 500 million years ago^27^ and emphasizes the fundamental importance of Sara endosomes inheritance in cellular fate determination. The characterization of this mechanism allows to understand the neurogenesis and development of zebrafish central nervous system with potential translation in mammals and human biology.

## Methods

### Zebrafish strains and maintenance

Zebrafish strains husbandry was maintained as described in ref. ^28^ and in accordance of the Swiss Veterinary Service law. Embryos were grown at 28 °C and their stage monitored by counting somites number. All experiments were performed on AB zebrafish background. Zebrafish strains were produced by us or ordered from European Zebrafish Resource Center (Supplementary Table 1).

### Database research and sequences

To find Patronin homologs in Zebrafish, BLAST (NCBI) of Patronin protein sequence (NCBI: ALT55646) was used against the Zebrafish proteome (taxon: 7955). Significant alignments were found for the different CAMSAP proteins: CAMSAP1a (NCBI: NP_001159727), CAMSAP1b (NCBI: NP_001093471), CAMSAP2a (NBCI: NP_00103846), CAMSAP2b (NCBI: XP_009297074), CAMSAP3a (NCBI: XP_021330421), CAMSAP3b (NCBI: XP_003197845). Same methodology was used to find Klp98A (NCBI: Q9VB25) homolog: KIF16Ba (NCBI: XP_009292601). Each protein sequences were run into SMART to predict CAMSAP domains (Fig. S6a).

### cDNA, plasmids and primers

To obtain open reading frames of *CAMSAP2a*, *CAMSAP3a*, *Pard3ab*, *Sara*, and *KIF16Ba*, a cDNA library was generated (SuperScript IV First-Strand Synthesis System, ThermoFisher) from 5dpf zebrafish embryos (Trizol reagent, Invitrogen). AscI and FseI restriction enzymes were used to insert coding sequences in “pCS2 + Fluorophore” plasmids having a sp6 promoter site to produce mRNA. Other plasmids were ordered on AddGene (https://www.addgene.org/) and modified to clone the sequences of interest into pCS2 backbone (Supplementary Tables 2 and 3).

### mRNA and morpholino injections

mRNA was in vitro transcribed using Sp6 polymerase (mMachine Invitrogen kit, ref. AM1340). A PV-820 Pico-injector (World Precision Instruments) and a Narashige micromanipulator were used for microinjection. Every injection was performed in one-cell-stage embryos, except for mScarlet-CAMSAP3a, Par3-mCherry and pSMOrange mRNA which were injected at 32-cell-stage to obtain a mosaic expression.

ATG morpholinos targeting RNA starting sequences were designed and ordered from GenTools (Supplementary Table 4). Control MO were designed with 5 mismatches from the original sequence. Morpholino injections were done in one cell stage embryos. Injected quantities were adjusted to have the highest possible concentration without severe toxicity (range from 0.5 to 2 ng per injection).

Rescue experiments for the spindle-MT asymmetry decrease observed in *CAMSAP2a*^*-/-*^*; CAMSAP3a*^*MO*^ 24hfp Zebrafish (Fig. 5e) were performed by injection of mix containing either *CAMSAP3a* MO (1.5 ng) + mCherry-CAMSAP2a mRNA (1 ng) + GFP-DCX mRNA (1 ng) or *CAMSAP3a* MO (1.5 ng) + mScarlet-CAMSAP3a mRNA (1 ng) + GFP-DCX mRNA (1 ng) in one-cell-stage *CAMSAP2a*^*-/-*^ mutant embryos. Rescue experiment for Sara endosome asymmetric inheritance in *KIF16Ba* MO embryos (Fig. S5a) was performed by injection of a mix containing *KIF16Ba* MO (0.8 ng) + KIF16Ba mRNA (1 ng) + mCherry-Sara mRNA (1 ng) in one-cell-stage *GFP-DCX* transgenic embryos. Then 24hpf injected zebrafish were screened for positive GFP and mCherry/mScarlet dual expressions and mounted for microscopy.

### Generation of *CAMSAP* CRISPR knock out

sgRNA targeting *CAMSAP2a* coding sequence was selected with the help of CRISPR Scan^29^ (https://www.crisprscan.org/) to prevent off targeting. sgRNA targeting *CAMSAP3a* was designed and produced at Merck (Merck sgRNA service) following the same rules of selection as *CAMSAP2a* sgRNA (Supplementary Table 5). Afterward, one-cell-stage AB zebrafish embryos were injected with 1 nl calibrated drop containing: 1.5 µl of sgRNA (0.5 µg/µl), 1.5 µl of Cas9 protein (5 µg/µl, ThermoFisher, ref. A50576) and 2 µl of nuclease free water. Mutant zebrafish were identified by genotyping (Sanger sequencing, https://www.fasteris.com/en-us/) to select deletion mutations leading to a frameshift of the coding sequence and premature stop codon (Fig. S6e, f). Identified mutants were crossed through multiple generations until reaching heterozygosity, homozygosity, or combinations of mutations. Combination of *CAMSAP2a*^*-/-*^ and *CAMSAP3a*^*-/-*^ mutations led to fish death. Therefore, double mutants were kept heterozygous and embryos resulting from their cross were sequenced at 24hpf with a ZEG device^30^ and sorted according to genotype.

### Generation of *GFP-Sara* CRISPR knock in

The technology used to obtain a knock-in in *Sara* gene was inspired by D. Grunwald work^31^. pKHR5 plasmid^31^ was modified to insert a GFP in front of *Sara* exon 2. A FLP removable mVenus sequence expressed under the alpha crystallin promoter *CryA*, allowing to sort the well injected embryos by looking at mVenus expression in the retina of embryos from 48 hpf on was also inserted. The following mix was injected (1 nl drop) in one-cell-stage embryos: 2.5 µl of linearized donor plasmid (100 ng/µl), 1 µl of sgRNA (1 µg/µl), 0.6 µl of Cas9 (5 µg/µl, ThermoFisher, ref. A50576), 0.5 µl of phenol red and 0.4 µl of nuclease free water. 48hpf injected embryos having green retina were analyzed by PCR, grown as F0 and in-crossed in groups (Supplementary Table 6). Then, resulting F1 embryos having a green retina were amplified inside and outside of the donor plasmid and crossed with WT embryos. When F3 homozygote zebrafish were obtained, a western blot analysis was performed on 5dpf embryos to show the presence of GFP-Sara using a mouse anti GFP antibody (Roche, ref. 11814460001) (Fig. S6l). Input loaded volume was 10 µL (10/1,000 µl) and IP loaded volume was 10 µL (10/35 µl). Gel was exposed during 5 min.

### Stereomicroscope

Embryos were first dechorionated in fish water medium with 0.003% of Tricaine (Sigma, ref. A5040) to anaesthetize them. Then, they were imaged with a Leica Stereomicroscope M80 equipped with a Leica IC80 HD camera. Somites numbers of individual embryo were manually counted to assess embryonic developmental stage.

### Embryo mounting

Embryos were first dechorionated in fish water medium with 0.003% of Tricaine to anaesthetize them. Then, they were mounted in 1% low-melting point agarose (Sigma, ref. A9414) with the spinal cord close to the coverslip.

### Spinning disk confocal microscopy

Embryos were imaged on a 3i Marianas spinning disk confocal setup based on a Zeiss Z1 stand with a x63 PLAN APO NA 1.4 oil immersion objective. Intensity of the laser was adjusted to avoid bleaching. Division of neural precursor cells are acquired in the 6–8 somite area of zebrafish spinal cord on 10–13 µm depth with z-stacks of ΔZ = 0.8 µm and Δt = 15 s until the end of the cytokinesis.

### Image analysis

5D hyper stack images (3D + Time + Channel) were exported in Tiff files from SlideBook 6.0 software. Then, images were treated with ImageJ and Matlab software. Custom written codes (available upon request) were used to acquire and process the data.

### Sara endosomes ratio quantification

Sara endosomes ratios were quantified using injection of mRNA coding for mCherry-Sara. Dividing NPs in the 6–8 somite area of Zebrafish spinal cord were imaged (Fig. 1e). Z stacks containing all the visible dividing cell (Δz = 0.8 µm, depth = 8 µm) at *t* = 75 ± 30 s (see time registration) were projected using sum intensity projection. Two areas on both daughter cells and a third area in the cytosolic background were drawn and saved as regions of interest (ROI). Then a custom ImageJ code uses the third ROI to subtract background intensity and calculates Sara endosomes ratio in pole A as follow:

$${Ratio}\,{of}\,{Sara}\,{endosomes}\,{in}\,{pole}\,A=\frac{{Total}\,{Intensity}\,{ROI}\,{pole}\,A}{{Total}\,{Intensity}\,{ROI}\,{pole}\,B}$$

### Spindle microtubule enrichment quantification

Spindle-MT densities were quantified using GFP-DCX as marker of MTs. Except for analysis of *CAMSAP* mutant fish where GFP-DCX mRNA was injected, the transgenic *GFP-DCX* zebrafish strain was used. The same acquisition parameters and methodology as Sara ratio quantification were used. Spindle-MT enrichment in pole B was calculated as follow:

$$ 	 {spindle}\,{MT}\,{enrichment}\,{in}\,{pole}\,B\\ 	 = \frac{{Total}\,{Intensity}\,{ROI}\,{pole}\,B-{Total}\,{Intensity}\,{ROI}\,{pole}\,A}{{Total}\,{Intensity}\,{ROI}\,{pole}\,A}{{{{{\rm{\times }}}}}} \times 100$$

The normalized enrichment of spindle-MT density in pole B: Δ, was calculated with the following equation:

$$\Delta =\frac{{Total}\,{Intensity}\,{ROI}\,{pole}\,B-{Total}\,{Intensity}\,{ROI}\,{pole}\,A}{{Total}\,{Intensity}\,{ROI}\,{pole}\,B+{Total}\,{Intensity}\,{ROI}\,{pole}\,A}$$

To measure spindle-MT enrichment in *CAMSAP* mutants, *CAMSAP2a*^+/−^; *CAMSAP3a*^*+/−*^ zebrafish were incrossed to produce variety of mutant embryos. One-cell-stage embryos were injected with GFP-DCX mRNA and grown at 28 °C for one day. Then, 24hpf embryos with unknown genotype were mounted for microscopy to image and quantify spindle-MT enrichment. Later, embryos were unmounted and sequenced to assign measured spindle-MT enrichments with the different genotypes indicated in Figs. 5e and S6g.

### CAMSAP2a and CAMSAP3a ratios quantifications

CAMSAP2a and CAMSAP3a ratios were quantified using mCherry-CAMSAP2a and mScarlet-CAMSAP3a mRNA overexpression in one-cell-stage embryos and 32-cell-stage embryos respectively (Fig. 5a, c). The same acquisition parameters as Sara ratio quantification and methodology were used. The ratio of CAMSAP2a/CAMSAP3a was calculated as follow:

$${CAMSAP}\,{ratio}\,{in}\,{pole}\,B=\frac{{Total}\,{Intensity}\,{ROI}\,{pole}\,B}{{Total}\,{Intensity}\,{ROI}\,{pole}\,A}$$

### Par3 ratio quantification

Par3 ratios were quantified using overexpression of Par3-mCherry mRNA in 32-cell-stage embryos (Fig. S1e). Note that only the cortical expression of Par3 was quantified, therefore its cytoplasmic expression was not considered to calculate the ratio. The same acquisition parameters as Sara ratio quantification and methodology were used. Par3 ratio in cell B was calculated as follow:

$${Par}3\,{ratio}\,{in}\,{cell}\,B=\frac{{Total}\,{Intensity}\,{ROI}\,{cell}\,B}{{Total}\,{Intensity}\,{ROI}\,{cell}\,A}$$

### Clustering analysis

To cluster 1-dimension and 2-dimensions datasets, we used a custom Matlab algorithm based on the Akaike Information Criterion (AIC), which compares Gaussian Mixture Models (GMM) of different cluster numbers fitted by natural logarithm of the likelihood function. GMM models were compared for clustering dataset in 1 to 4 clusters. The model with the lowest AIC score corresponds to the most likely number of clusters in the population. Using this number of clusters, we then used two methods of clustering: K-mean clustering, which separates clusters according to their mean and GMM, which uses fitted gaussians to cluster the data. Finally, to measure the goodness of the clusters, we calculated their Dunn Index (DI), an algorithm to evaluate clustering based on mean and variance. A high DI indicates a reliable clustering.

### Tracking, spatial registration and time registration

The movements of mCherry-Sara positive endosomes on spindle-MTs (labeled by GFP-DCX) were tracked with two different acquisition parameters referred as “normal tracking” and “fast tracking”.

i. Normal tracking: Δz = 0.8 µm, depth = 3.2 to 6.4 µm and Δt = 15 s for endosome and MT channels.
ii. Fast-tracking: Δz = 0.5 µm, depth = 3 µm and Δt ~ 0.75 s (1.35hz) for endosome channel and, Δz = 0.5 µm, depth = 3 µm and Δt = 15 s for MT channel.

In order to have a centrosome-to-centrosome horizontal axis, the dividing NPs were rotated on ImageJ before treatment. Trackmate plugin was used in ImageJ to track Sara endosome movements^11^. Trackmate Laplacian of Gaussian (LoG) filter was applied on Sara endosome channel to detect 1 µm diameter objects. Quality filter, based on local maxima value and closeness to the specified diameter was manually applied on each time point to discard low quality objects. Then, Trackmate Simple Lap Tracker was used to establish Sara endosome tracks. “Linking max distance” and “Gap closing max distance” parameters were set to 2.7 µm for “normal tracking” and 2 µm for “fast tracking”. Those values are based on Sara endosomes size (~1 µm) and velocity in fly^2^ ($v({fly})=0.173\pm 0.007{{{{{\rm{\mu }}}}}}m.{s}^{-1}$) which is linked to the maximum traveling distance for an endosome per time frame. “Gap closing max frame gap” value was set to 4 frames. No Trackmate filter was applied on tracks. Sara endosome data generated by Trackmate are *x* coordinate, *y* coordinate, time frame, mean intensity, diameter and signal to noise ratio (SNR) computed as ${SNR}=\frac{{Iin}-{Iout}}{{stdin}}$, where ${Iin}$ is the mean intensity inside the spot volume, ${Iout}$ is the mean intensity in a ring ranging from its radius to twice its radius and ${stdin}$ is the standard deviation computed within the spot. In addition, the GFP-DCX channel was used to manually record the *x* and *y* coordinates of centrosome in pole B (α), centrosome in pole A (ƴ) and cell center (β) for each time point (Fig. S2a, b). It is worth disclaiming that it was difficult to manually assign a precise position for β before appearance of the cleavage furrow. Therefore, a correction was applied on the manually recorded β coordinate to have $\Vert \overrightarrow{\alpha \beta }\Vert =\Vert \overrightarrow{\beta \gamma }\Vert$ during metaphase and anaphase until clear appearance of the cleavage furrow defined as cell center. The coordinates of α, β and ƴ were used for the spatial registration of Sara endosome coordinates in the “normal tracking dataset” and “fast tracking dataset”.

To do the spatial registration, Sara endosome coordinates (E1), were considered as the orthogonal projection of the location of an endosome into the line connecting either centrosome α or ƴ with the spindle center β set as origin. The orthogonal length between the endosome location and projection was set as new *y* coordinate and the length between the endosome projection and β was set as new *x* coordinate (Fig. S2b). According to the sign of the new *x* coordinate, Sara endosomes were attributed to pole B (*x* < 0) or pole A (*x* > 0). Sara endosomes located at the undefined overlapping region around β were not considered after appearance of the cleavage furrow. In addition, Trackmate generated data were filtered to not consider diameter values below 0.1 diameter quantile, SNR values below 0.1 SNR quantile and $\Vert \overrightarrow{E1{{{{{\rm{\beta }}}}}}}\Vert$ > 6 µm.

It is worth noting that this spatial registration considers Sara endosome coordinates in 2D (from a maximum projection of the movie having the same ΔZ above and below cell center). However, the coordinates of centrosome in pole B (α), centrosome in pole A (ƴ) and cell center (β) are not perfectly co-planar (their *z* coordinates are not exactly the same) and this might introduce some imprecisions in the determination of their distances in 2D. We estimated that the maximum angle between the three coordinates was 32° for a 6.4 µm depth movie, introducing a *x* coordinate error of 0.93 µm at the edge of the cell and 0.19 µm at the central spindle. Since in the density analysis (see below) bins are of 0.5 µm, these errors are negligible.

Time registration was only applied to the “normal tracking dataset”. We noticed that, in metaphase, α, β and ƴ are aligned very precisely (the angle $\widehat{\alpha \beta \gamma }$ is close to 180°), while, coinciding with the formation of the cytokinetic furrow, α, β and ƴ depart from this alignment (Fig. S2c–f). We defined *t* = 0 s as the time corresponding to the frame preceding the dealignment event (angle $\widehat{\alpha \beta \gamma }$ decrease >10°, manually verified) induced by the formation of the cytokinetic cleavage furrow. As a consequence of registering time this way, the $\widehat{\alpha \beta \gamma }$ (*t*) traces collapse to an angle close to 180° before *t* = 0 s and, after that, the traces showing the dynamics of $\widehat{\alpha \beta \gamma }$ collapse into a single curve, showing that the timing and dynamics of dealignment with respect to the formation of the cleavage furrow is robust.

Then Sara endosomes data were treated following Derivery and al. methodology^2^ to calculate the physical parameters used in Eq. 1 (see below).

### Velocity $v$ analysis

The fast-tracking dataset (see above) of Sara endosomes movements was used to measure endosomal velocity along *x*-axis. A custom Matlab code was made to select segments within Sara endosome *x* motility tracks where endosomal movement is for at least 5 consecutive time points in the same direction. Then, each segments were plotted for *x* position *versus* time and fitted with a linear regression to obtain velocity. Mean velocity of each segments was computed and gave $v=\left(1.36\pm 1.07\right){10}^{-1}{{{{{\rm{\mu m}}}}}}\,{s}^{-1}$ (39 segments, *mean R*^2^ *fit* *=* 0.92 ± 0.049, 95% confidence).

### K_off_ and K_on_ρ analysis

To measure the off rate (${k}_{{off}}$) and on rate $({k}_{{on}}\rho )$ of Sara endosomes from MTs at central spindle, the fast-tracking dataset was used. First, the dataset was treated with a custom Matlab code to keep tracks within an area of 3 µm by 3 µm centered around β. An additional manual verification of the tracks was performed to discard bad quality tracks. Then “transport-segments” were identified among the selected tracks according to the following criteria:

i. instantaneous speed of Sara endosome must be higher than 0.15 µm.s^−1^. Since the calculated velocity and diffusion are *v* = (1.36 ± 1.07)10^−1 ^µm s^−1^ and *D* = (8.15 ± 0.46)10^−3^ μm^2^ s^−1^, this threshold decreases the probability to have a diffusion event in the “transport-segment”.
ii. the duration of a “transport-segment” must be at least three frames long.
iii. Sara endosome motility must be in the same direction for all the frames in the “transport-segment”.

After identification of “transport-segments”, the number of “transport-segments” as a function of their duration was plotted. Decay time of the exponential fit indicated$\,{k}_{{off}}=(0.97\pm 0.33){s}^{-1}$ (*R*^2^ = 0.99, 95% confidence; Fig. S4e).

Segments in between the “transport-segment” were called “diffusion-segments”. The number of “diffusion-segments” was binned (15 s bins) and represented as a function of their duration. Decay time of the exponential fit indicated ${k}_{{on}}\rho$ = (0.057 ± 0.004)s^−1^ (*R*^2^ = 0.99, 95% confidence; Fig. S4f).

### Mean square displacement analysis

To determine the Mean Square Displacement (MSD) of Sara endosome tracks, the normal-tracking dataset was used. Tracks were manually and automatically refined to keep tracks:

i. at least eight timeframes long
ii. with a maximum gap time of 45 s,
iii. and within an area of 10 µm by 9 µm centered around β.

Afterward, MSD analyzer package^32^ was used with a custom Matlab code to generate $MSD(t)=\langle (\varDelta {x}^{2})\rangle +\langle (\varDelta {y}^{2})\rangle$ of each individual track (Fig. S4c; S5b). Mean weighted ${MSD}\left(t\right)$ was computed using the number of tracks and the number of averaged points as weight. Then, the mean *MSD(t)* was fitted with the linear model: *MSD(t)* = 4*Dt* or the quadratic model: $MSD(t)=4Dt+{v}^{2}{t}^{2}$ (Fig. S4d and Fig. S5b, c). In control, the quadratic fit captures best the motion of endosomes (R^2^ = 0.99 for quadratic fit and R^2^ = 0.97 for linear fit, 95% confidence). In *KIF16Ba* morphant, the quadratic fit gives a complex value for *t* (as *t* ^2^ < 0) indicating confined diffusion. This was confirmed by an anomalous fit of *KIF16Ba* morphant MSD: ${MSD}(t)=4D{t}^{{{{{{\rm{\alpha }}}}}}}$ where α < 1 is found. Therefore, KIF16Ba is essential for the directed motility of Sara endosomes beyond diffusion and only the linear fit of *KIF16Ba* morphant MSD was considered. Diffusion values were extracted from the corresponding fitting models. The mean of diffusion values found in control NPs (quadratic model) and *KIF16Ba* morphant NPs (linear model) was used to determine the Diffusion parameter $D=\left(8.40\pm 0.42\right){10}^{-3}\mu {m}^{2}{s}^{-1}$ used in Eq. 1.

### Antiparallel central spindle length analysis

To quantify the length of the spindle-MT overlapping region, we used mCherry-MKLP1 overexpression in *GFP-DCX* transgenic Zebrafish embryos (Fig. S3g). Dividing NPs were imaged with the following parameters: Δz = 0.8 µm, depth = 8 µm and Δt = 15 s. mCherry-MLKP1 was only detectable after the appearance of the cleavage furrow and is located at the central spindle region, shrinking according to the contraction of the spindle-MTs. mCherry-MKLP1 length, $l=(1.3\pm 0.1){\mu m}$ (*n* = 3 NPs from 3 independent Zebrafish) was manually measured on ImageJ software using the line scan of a maximal z-projection at registered *t* = 30 ± 15 s.

### Quantification of Sara endosomes percentage at central spindle

To quantify Sara endosomes percentage at central spindle (Figs. 3g, 4g, 5f), the normal tracking dataset was used and refined with a custom Matlab code to keep tracks:

i. at least five timeframes long,
ii. with a maximum gap time of 30 s
iii. within an area of 10 µm by 9 µm centered around β

Sara endosomes were considered at central spindle if their absolute *x* normalized coordinate was inferior to 1 µm. Percentage of Sara endosomes at central spindle was calculated with the following equation:

$$	{Sara}\,{endosomes}\,{percentage}\,{at}\,{central}\,{spindle}\\ 	\quad =\frac{{Number}\,{of}\,{Sara}\,{endosomes}\,{at}\,{central}\,{spindle}}{{Total}\,{number}\,{of}\,{Sara}\,{endosomes}}\times 100$$

Rob Campbell, shadedErrorBar function was used on matlab to generate shades of the standard error. If Sara endosomes are homogeneously located in a dividing NP, their percentage at central spindle should be 20% because the 2 µm large region considered as the central spindle region represents 20% of the 10 µm large total region (Fig. S2a). To statistically evaluate the difference of Sara endosome percentage at central spindle, the data were divided into the homogenous (t from −285 s to −225 s), recruitment (t from −210 s to −105 s) and departure phases (t from −90 s to 150 s). Then a two-sample Kolmogorov–Smirnov test was used to compare phases between control and mutant/morphant NPs.

### Automatic quantification of Sara endosomes ratio

A custom Matlab code was used to automatically quantify Sara endosomes ratio according to mCherry-Sara total intensity in the normal tracking dataset. Data were sorted to keep tracks within an area of 10 µm by 9 µm centered around β. Afterward, Sara endosomes ratios were calculated for each time frame with the following equation:

$${Sara}\,{endosomes}\,{ratio}\,{in}\,{pole}\,A=\frac{{Total}\,{intensity}\,{of}\,{Sara}\,{endosomes}\,{in}\,{pole}\,A}{{Total}\,{intensity}\,{of}\,{Sara}\,{endosomes}\,{in}\,{pole}\,B}$$

Ratio above 1.5 or below 0.67 (1/1.5) were considered as an asymmetric inheritance of Sara endosomes in pole A or pole B, respectively.

### Heatmap of Sara endosomes density

To generate the spatiotemporal density plots (heatmaps, Fig. S3c, e, f; S5d; S7a; S8a), the normal tracking dataset was used and refined with a custom Matlab code to retain tracks (i) at least four timeframes long, and (ii) within an area of 10 µm by 9 µm centered around β. Then, the number of Sara endosomes were counted in bins of Δ*x* = 0.5 µm and Δt = 15 s (20 bins per time point) and referred as $N(x,t)$. Negative *x* values correspond to pole B having more spindle-MTs and positive *x* values correspond to pole A having less spindle-MTs.

Each $N(x,t)$ values were normalized to the total number of Sara endosomes in the dataset and displayed as heatmap with a Red Hot colormap lookup table (LUT).

To generate the randomized heatmap, we first calculated $\lambda (t)$ in the asymmetric dataset as:

$$\lambda (t)=\frac{\mathop{\sum }\nolimits_{x}^{20}{{{{{\rm{N}}}}}}(x,{{{{{\rm{t}}}}}})}{20}$$

Then, for each time point, we generated 20 values from a Poisson distribution around $\lambda (t)$ corresponding to randomized Sara endosome bin numbers. Each one of the 20 generated values was randomly assigned to a bin for each time point and colored with the Red Hot colormap LUT to display the randomized heatmap of Sara endosomes density. This randomized heatmap was used to compare the distributions of Sara endosomes densities coming from experimental data (Fig. S3c, d).

### Poisson statistics and distribution

For each dataset, the expected Poisson statistics around λ(t) were calculated for each $N(x,t)$ values according to the following equation:

$${P}_{{bin}(x,t)}=\frac{{\lambda (t)}^{{{{{{\rm{N}}}}}}(x,t)}\times {e}^{\lambda (t)}}{N(x,t)!}$$

Then, ${P}_{{bin}(x,t)}$ values were normalized by dividing them with the maximum ${P}_{{bin}(x,t)}$ value of the time point. A custom LUT was applied to visualize the normalized ${P}_{{bin}(x,t)}$ values, with red colors corresponding to a high number of endosomes, white colors to a number of endosomes close to λ(t) and blue colors to a low number of endosomes (Fig. S3a). Gradient of the custom LUT was adjusted for each time point to go from λ-3 $\sqrt{{{{{{\rm{\lambda }}}}}}}$ (blue) to λ + 3 $\sqrt{{{{{{\rm{\lambda }}}}}}}$ (red) with $\sqrt{{{{{{\rm{\lambda }}}}}}}$ corresponding to the standard deviation of λ.

### ANOVA analysis for density comparison

To compare the effect of cell side location or cell center location on Sara endosomes mean densities, a statistical ANOVA analysis was performed. For each time point, $N\left(x,t\right)$ values were segmented according to their location: pole B (*x* from −5 μm to 0 μm), pole A (*x* from 0 μm to 5 μm), cell sides (*x* from −5 μm to −1 μm and 1 μm to 5 μm) and cell center (*x* from −1 μm to 1 μm). Then segments were compared by ANOVA analysis to obtain a *p*-value rejecting (*p* < 0.05) or confirming (*p* > 0.05) the hypothesis that the means of the segments is the same.

To further analyze the dynamic of ANOVA *p*-values in a dataset, we randomly shuffled the twenty $N\left(x,t\right)$ values of each time bin of a dataset and performed the same statistical ANOVA analysis as described above. We repeated this methodology 10,000 times and established the frequency of having a similar dynamic of the *p*-values compared to the *p*-value dynamics observed in the different datasets (Fig. 3e, f). For comparison of the ANOVA *p*-value dynamic in pole B *versus* pole A mean Sara endosomes densities of the control asymmetric shuffled dataset, we found that 1.4% of the 10 000 randomly shuffled datasets had: no *p*-value < 0.05 before *t* = 0 s and four *p*-values < 0.05 after *t* = 0 s. This low percentage indicates that the pattern observed in the *p* value dynamic of the asymmetric dataset is not coming from a random distribution. For comparison of ANOVA *p*-value dynamic in pole B *versus* pole A mean Sara endosomes densities of the control symmetric dataset and randomized dataset (from symmetric dataset), we found that 75% and 84% of the respective randomly shuffled datasets had at least one time point with a *p*-value < 0.05. This similarity of the 75% and 84% frequencies indicates that the *p*-value dynamic of the control symmetric dataset is comparable to the *p*-value dynamic of the randomized dataset and the appearance of a single, sporadic statistically significant time point is expected to happen by chance.

### Photoconvertible probe and lineage

mRNA coding for pSMOrange^10^ was injected in 32-cell-stage *GFP-DCX* transgenic embryos (Fig. 2a, b). Embryos were grown in the dark until reaching 27 ± 1 somite stage. pSMOrange is expressed in the cytosol and its mosaic expression allows to precisely target isolated dividing NP. A Phasor system (3i) was used to illuminate a 4 µm circular region (4 µm depth, ΔZ = 1 µm) with 2 × 30 pulses of 2% 405 nm UV laser resulting in a photoconversion of pSMOrange. Note that precise calibration and optimization of the microscopy system must be done to properly photoconvert a cell without damaging it. After photoconversion, the region around, above and below the photoconverted cell was scanned for undesired cells and bleached with a 100% intensity 543 nm laser to keep only the cell of interest. Then, embryos were released from agarose and kept in dark for 48 h at 28 °C in a 0.003% phenylthiourea (PTU) solution to stop pigmentation. Finally, embryos were mounted and imaged again to find back the photoconverted cell and its lineage (Fig. 2c, d).

### Statistics and reproducibility

All statistics were calculated using Matlab software with custom codes adapted from MathWorks functions (available upon request). All statistical test significances were considered using an α of 0.05. No statistical methods were used to predetermine sample size. R² fit values were determined with 95% confidence interval (Figs. 3i; 4f; Fig. S4d–f; S5c). ANOVA were made using one-way ANOVA (Figs. 3d–f; 4e, h; 5i; Fig. S3h, i; S7c; S8c). A two-samples Kolmogorov-Smirnov test (95% confidence) was used to compare distribution of data (Fig. 4g). Chi-square tests (95% confidence) were used to compare percentages of asymmetric NPs (Fig. 5E; Fig. S6d) and percentages of lineages (Fig. 2d; 5j). The following *p*-values from Chi-square tests were found: Fig. 2d *=0.0402 and **= 0.0011; Fig. 5e *= 0.0174 and **=0.0014; Fig. 5j *=0.0127; Fig. S1i *=0.0402 (all *vs* n.p), *=0.0145 and **=0.0021; Fig. S6d *=0.0401. Non indicated comparisons are non-significant. A log10 transformation was applied to the Sara ratio dynamic analysis (Fig. 3h; 4d; 5h). AIC, GMM, K-mean and Dunn-Index analysis were made using Matlab functions (MathWorks). A maximum of 3 NPs per embryos were imaged, apart for the lineage analysis where 1 NP per embryo was imaged. The experiments were not randomized and the investigators were not blinded to allocation during experiments, outcome assessment and analysis of the data.

### Reporting summary

Further information on research design is available in the Nature Portfolio Reporting Summary linked to this article.

### Supplementary information

Supplementary Information

Description of Additional Supplementary Files

Supplementary Data 1

Reporting Summary

## Data Availability

All data supporting the findings of this study are available within the paper, Supplementary Information for Supplementary Figs. and Supplementary Tables, and Supplementary Data 1 for data point values and raw images used for analysis. All other data are available upon request.

## Code availability

Custom codes used for analysis were made on Matlab 2021b and are available upon request.
