# Supplementary material for: Microtubule polarity determines the lineage of embryonic neural precursor in zebrafish spinal cord
Source: Commun Biol. 2024 Apr 10;7:439. doi: 10.1038/s42003-024-06018-7 (PMC11006876; doi:10.1038/s42003-024-06018-7)
Supplement: Supplementary file 1 — Supplementary Information [file 42003_2024_6018_MOESM1_ESM.pdf]

Supplementary Data

Microtubule polarity determines the lineage of embryonic neural precursor in zebrafish spinal cord

Clément-Alexis Richard[1], Carole Seum[1] & Marcos Gonzalez-Gaitan[1]

[1]Department of Biochemistry, Faculty of Science, University of Geneva, 30 Quai Ernest Ansermet, Geneva 1211, Switzerland

Supplementary Figures:

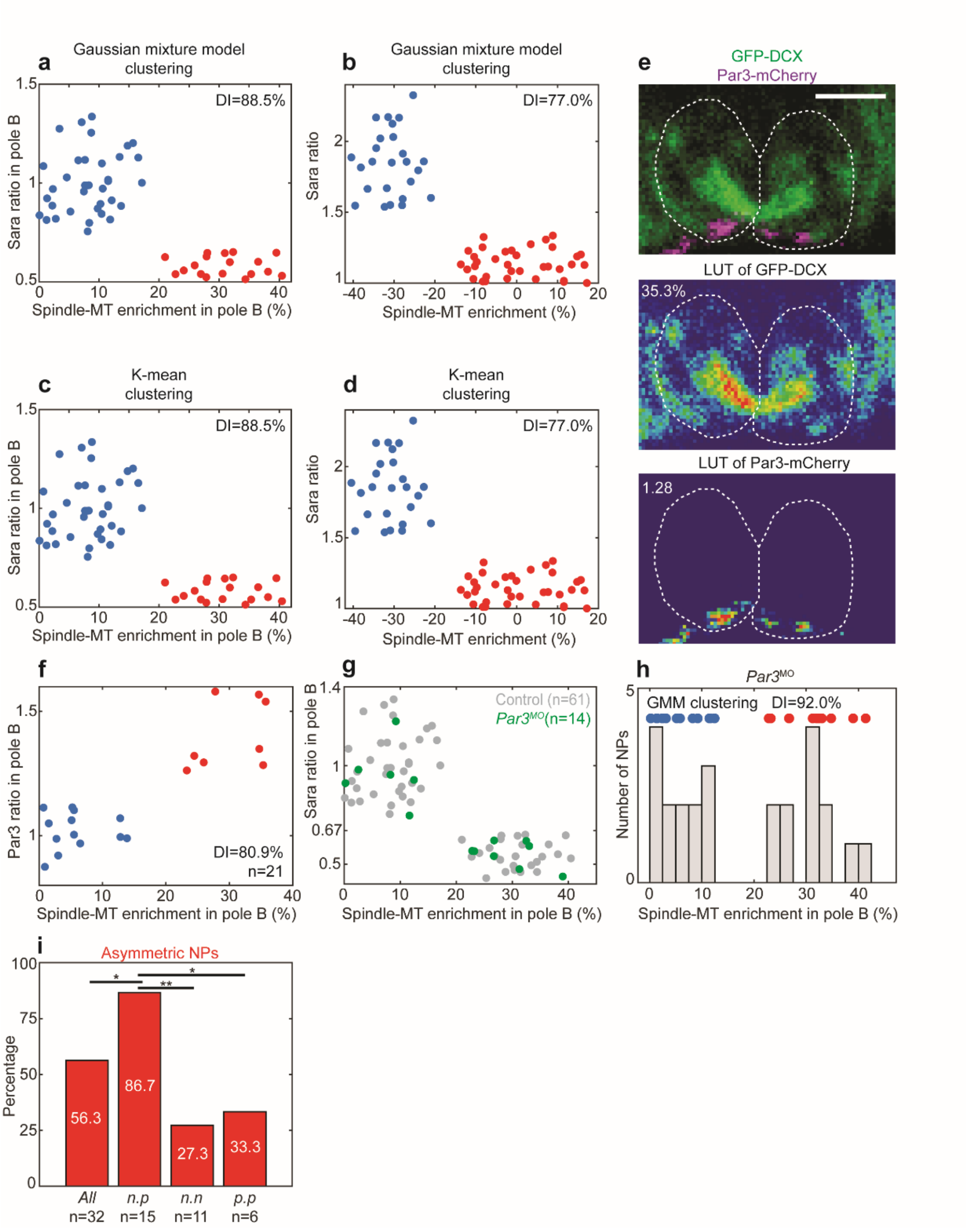

Figure S1: Clustering methodology and Par3 asymmetry. **a**-**d** GMM (**a**-**b**) or K-mean clustering (**c**-**d**) of Sara endosome ratio as a function of spindle-MT enrichment (n=62 NPs). **a**, **c** Sara endosome ratio and spindle-MT measured in pole B. **b**, **d** Sara endosome ratio and spindle-MT measured in the pole having more Sara endosome. **e**, Sum z-projection showing spindle-MTs (GFP-DCX, green) and Par3 (Par3-mCherry, magenta). LUT shows respective densities. Relative pole B percentage of spindle-MT enrichment and Par3 ratio are indicated. Dashed lines, cell contours. Bar, 5µm. **f**, GMM clustering of relative pole B Par3 ratio as a function of spindle-MT enrichment (n=21 NPs, DI=80.9%). Two clusters are found for symmetric (blue) and asymmetric (red) NPs. Note that in this report we focus on cortical Par3, and do not consider the cytoplasmic pool as studied in [17]. **g**, Relative pole B Sara endosome ratio as a function of spindle-MT enrichment for control (grey, n=61 NPs) and *Par3^{MO}* (green, n=14 NPs) datasets. *Par3^{MO}* dataset can be clustered in two NP pools (DI=85.7%). **h**, Histogram of spindle-MT enrichment measured in pole B of *Par3^{MO}* NPs (n=25). Above, data are clustered (GMM clustering analysis) into two groups (DI=92.0%), symmetric (blue dots) and asymmetric (red dots) NPs. **i**, Percentage of asymmetric NPs (according to spindle-MT enrichment) for *all* photoconverted NPs (n=32), *n•p* (n=15), *n•n* (n=11) or *p•p* lineages (n=6). *, p< 0.05; **, p<0.01. Non indicated comparison, non-significant (N.S).Chi-square tests, 95% confidence.

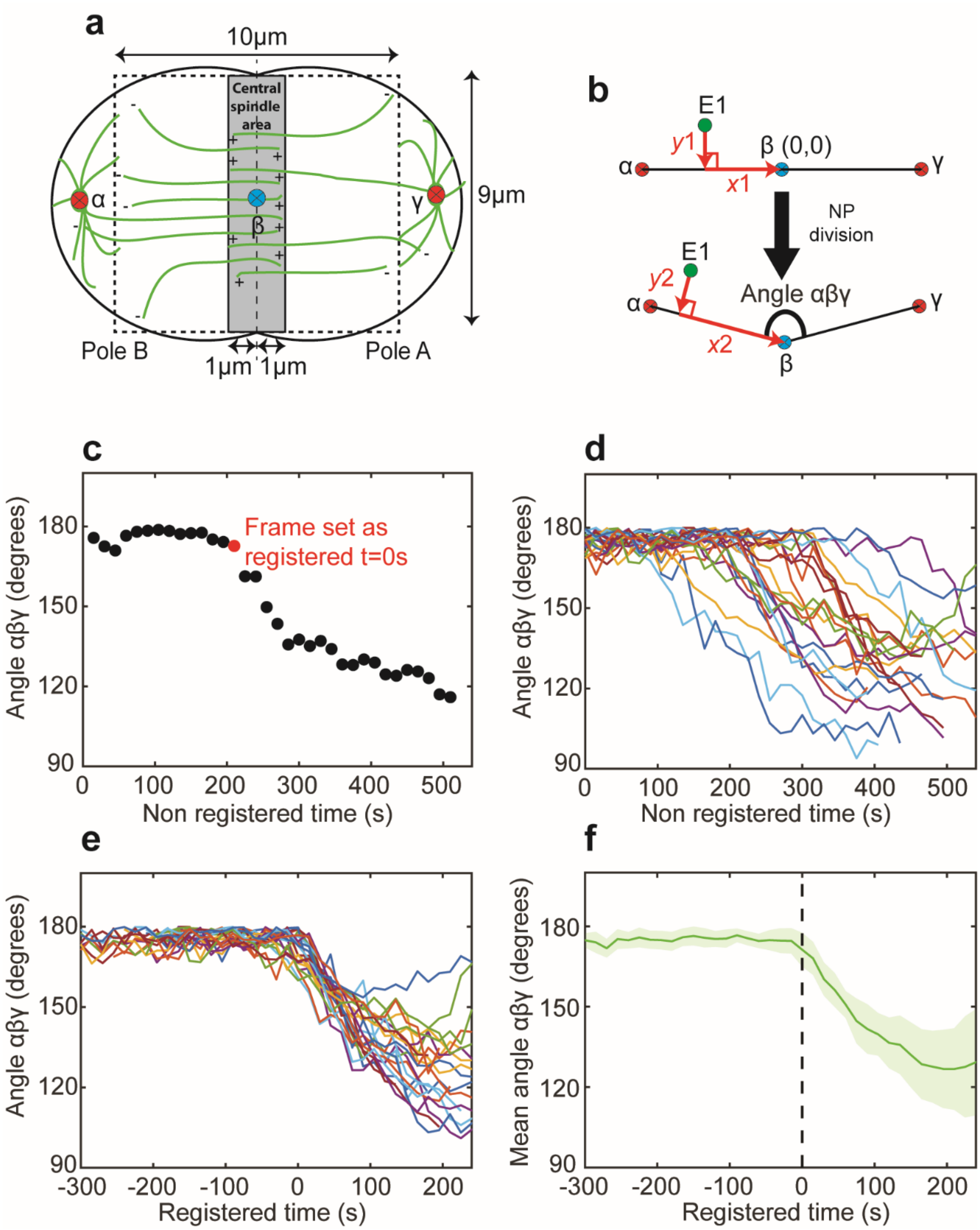

Figure S2: Space and time normalization of Sara endosome tracking. **a**, Scheme of a dividing NP indicating α, γ and positions β (centrosomes and spindle center, respectively). Grey region, central spindle area with antiparallel MT array. Dash box, Sara endosome tracking area. Green, spindle-MTs and their orientation (+ and – ends). **b**, Scheme of Sara endosome spatial registration. E1, Sara endosome position. Red arrows, orthogonal projection of E1 location on the line connecting either centrosome α or γ with the spindle center β set as origin. The orthogonal length between E1 and its projection is set as new *y* coordinate and the length between E1 projection and β is set as new *x* coordinate. The angle $\widehat{\alpha\beta\gamma}$ is used for time normalization of Sara endosome tracks. **c**, Methodology of time registration with registered t=0s set as the time preceding $\widehat{\alpha\beta\gamma}$ important decrease (>10°) (red dot). **d**-**f**, Traces of angles $\widehat{\alpha\beta\gamma}$ (**d**-**e**; n=23 NPs) or their mean (**f**) as a function of non-registered time (**d**) or registered time (**e**- **f**).

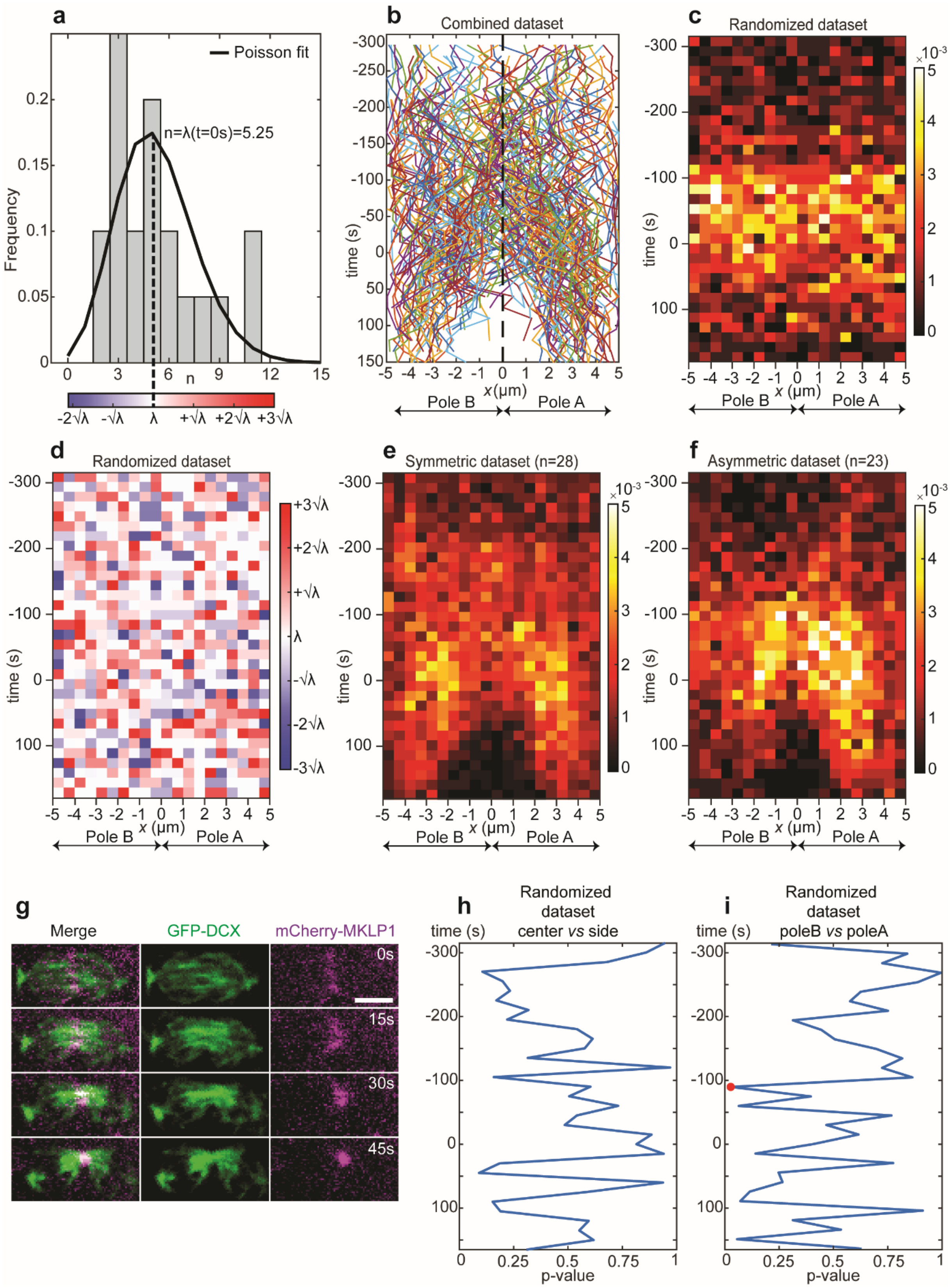

Figure S3: Density plots and randomized dataset . **a**, Histogram showing the frequency of Sara endosome number (n) per bin (bin Δ*x*=0.5μm, 20 bins per time point, see methods) at registered time t=0s from asymmetric heatmap dataset (**f**). Black line, fit of expected Poisson statistics around λ(t=0s) (mean number of endosomes per bin). Below, custom density LUT based on $\sqrt{\lambda}$ scale units showing high number of endosomes (red), number of endosomes close to λ (white) and low number of

endosomes (blue). Value of λ(t=0s) is indicated. **b**, Sara endosome *x*-axis individual traces as a function of registered time for combined dataset (n=356 tracks from 51 NPs). Dashed line, registered cell middle. **c**, **e**-**f** Spatio-temporal density plot of Sara endosome binned number as a function of registered time for randomized (**c**; see methods for randomized dataset generation), symmetric (**e**) and asymmetric (**f**) datasets. Individual binned numbers are normalized by the total number in the dataset, respectively. LUT indicates normalized densities of Sara endosomes from low (black) to high (white) densities. **d**, Sara endosome densities in space and time for randomized dataset as in Fig. 3b, c. **g**, Maximal z-projection time lapse of a dividing NP showing spindle-MTs (GFP-DCX, green) and central spindle antiparallel region labelled by mCherry-MKLP (magenta). Scale bar, 5µm. t corresponds to registered time. **h**, **i** ANOVA comparison of Sara endosome mean densities as a function of registered time between cell center and cell sides (**h**) or poleB and poleA (**i**) for randomized dataset as in Fig. 3d-f.

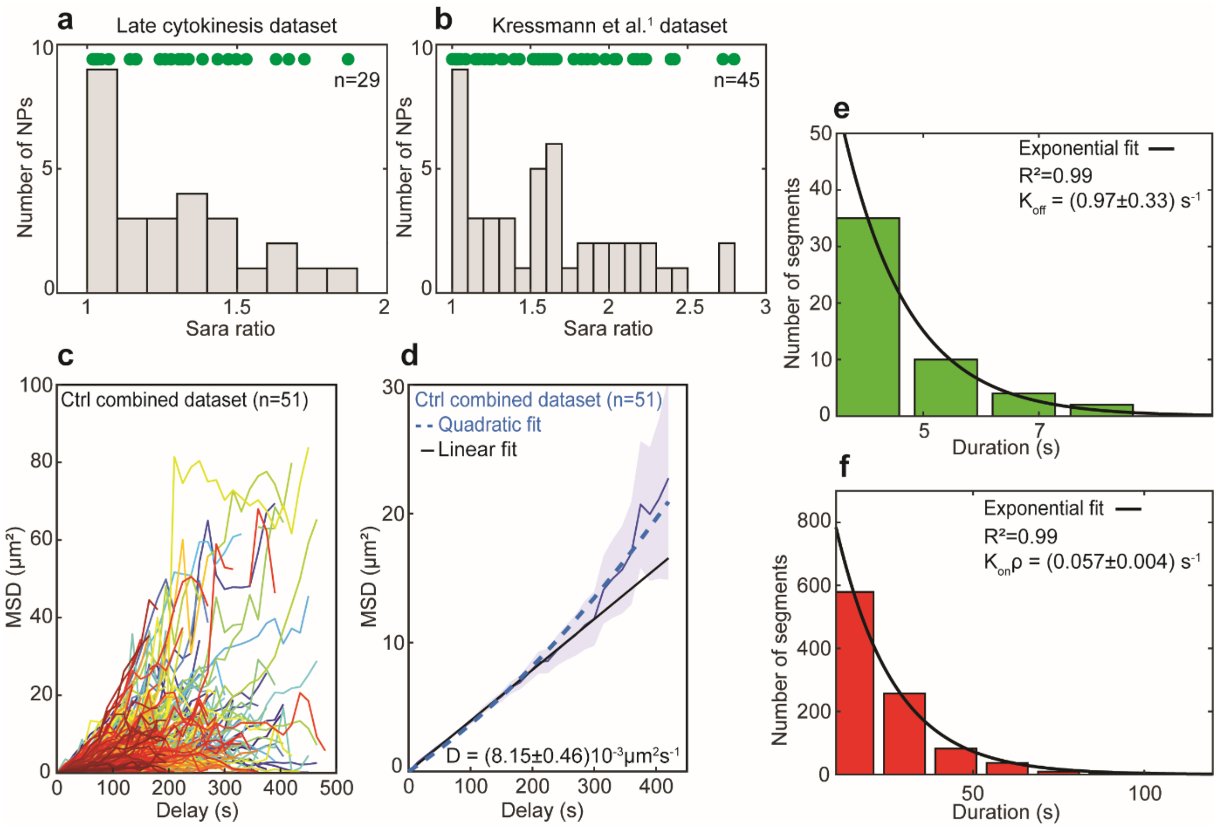

Figure S4: NP clusters merge after cytokinesis and methodology of acquisition for physical parameters of Sara endosome motility. **a**-**b**, Histograms of Sara endosome ratio measured in the pole having more Sara endosomes for late cytokinesis (**a**; n=29 NPs) or Kressmann et al.[1] (**b**; n=45 NPs) datasets. Above, clustering of the data merges (green dots), and NPs cannot be clustered in symmetric/asymmetric pools as in Fig. 1d. **c**, Individual MSD traces as a function of delay for each Sara endosome track in combined dataset (n=51 NPs, 337 tracks). **d**, Weighted average MSD as a function of delay for control combined dataset (blue line). Blue dashed line, quadratic fit. Black line, linear fit. Value of diffusion (D) calculated from the quadratic fit (R^2=0.99, 95% confidence, see methods) is indicated. Shade, SEM. **e**-**f**, Histograms of the distribution of duration of segments on transport (**e**; n=51 segments) and diffusive state (**f**; n=1009 segments) (see methods). Black line, exponential fit. Estimation of k_{off} and $k_{on}\rho$ from the exponential fits and their correlation coefficient (R^2, 95% confidence) are indicated.

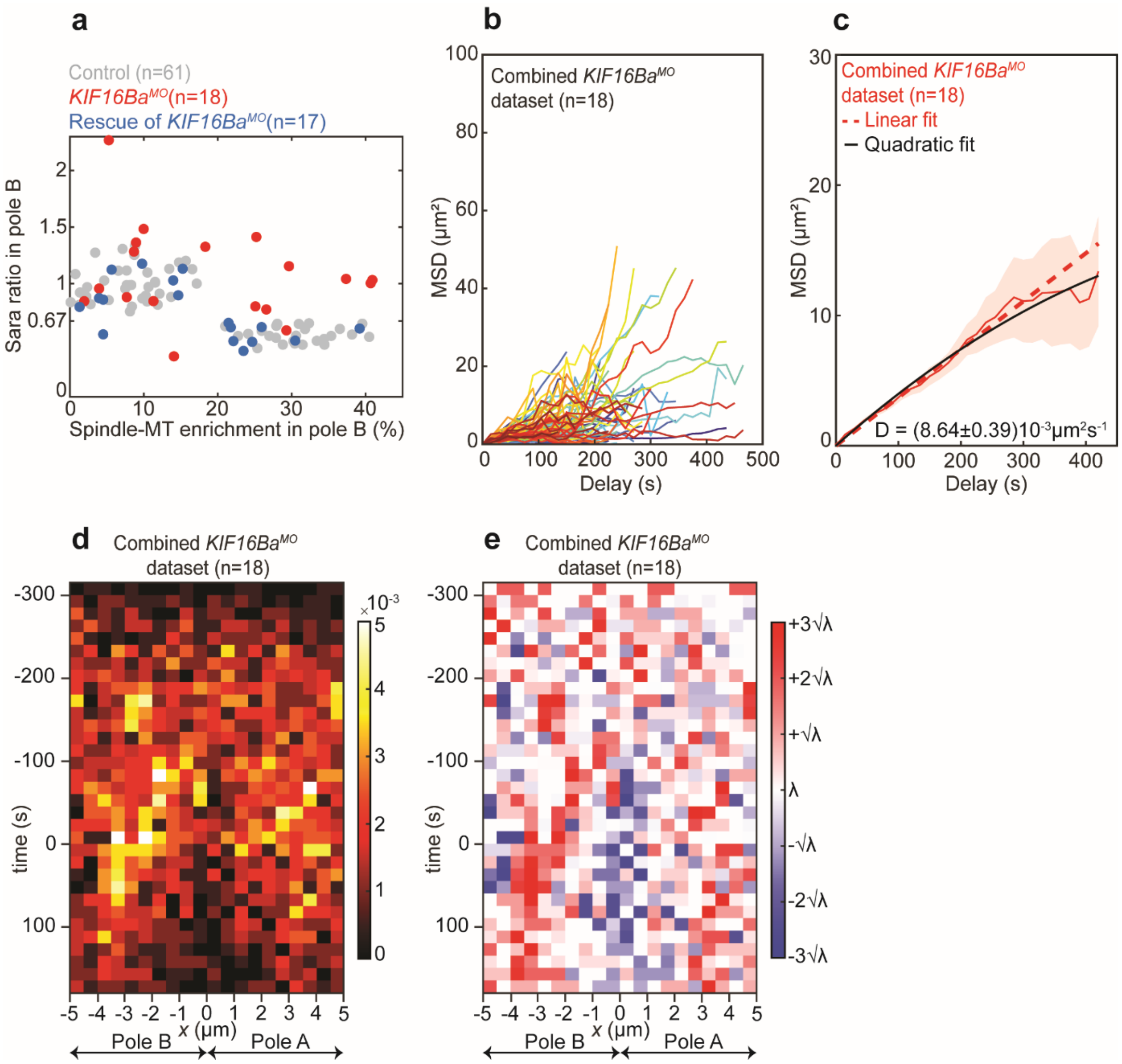

Figure S5: Rescue, MSD, Heatmaps and ANOVA for KIF16Ba^{MO} dataset: **a**, Relative pole B Sara endosome ratio as a function of spindle-MT enrichment for control (grey, n=61 NPs), *KIF16Ba^{MO}* (red, n=18 NPs) and rescue of *KIF16Ba^{MO}* (blue, n=17 NPs) datasets. **b**, Individual MSD traces as a function of delay for each Sara endosome tracks in *KIF16Ba^{MO}* dataset (n=18 NPs, 79 tracks). **c**, Weighted average MSD as a function of delay for control *KIF16Ba^{MO}* dataset (red line). Red dashed line, linear fit. Black line, quadratic fit. Value of D calculated from the linear fit (R^2=0.98, 95% confidence, see methods) is indicated. Shade, SEM. **d**-**e**, Spatio-temporal density plot of Sara endosome binned number as a function of registered time for *KIF16Ba^{MO}* dataset as in Supplementary Fig. 3c, e-f (**d**) and Fig. 3b-c (**e**).

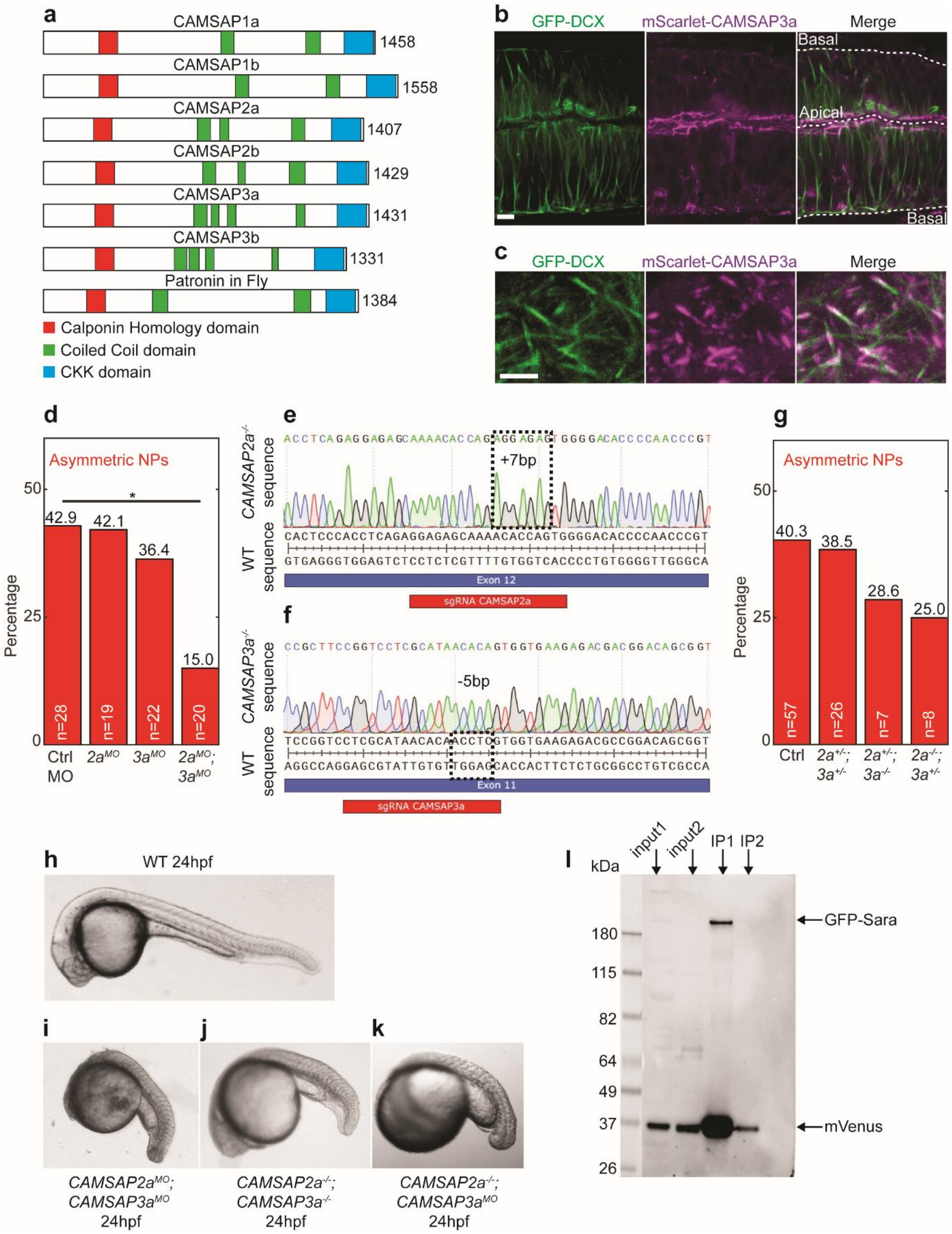

Figure S6: CAMSAP domains, morphants and mutants, and GFP-SARA CRISPR Knock-in: **a**, Scheme of SMART predicted domains for the different CAMSAP proteins found in zebrafish and comparison with fly Patronin (see methods for NCBI sequence IDs). CH domain (Red), Coil Coiled domains (green) and CKK domain (blue) are displayed from N-terminal (left) to C-terminal (right) of the corresponding protein with the relative number of base pairs indicated. **b**-**c**, Maximal z-projection showing CAMSAP3a (mScarlet-CAMSAP3a, magenta) and microtubules (GFP-DCX, green) in 24hpf zebrafish spinal cord (**b**)

or at dorsal cortex (**c**). Dash, apical and basal locations. Scale bars, 10µm (**b**), 5µm (**c**). **d**, **g** Percentage of asymmetric NPs in control and various *CAMSAP2a/CAMSAP3a* morphants (**d**) or mutants (**g**). Chi-square test, 95% confidence, * $p<0.05$. Other comparisons are N.S. **e**-**f**, Sequences of CRISPR knock out for *CAMSAP2a*$^{-/-}$ (**e**) and *CAMSAP3a*$^{-/-}$ (**f**) (Sanger sequencing). Below, corresponding wild-type (WT) sequences. Dash boxes, base pairs insertion (**e**) or deletion (**f**) following DNA cut and non-homologous end joining. Blue, exon targeted for CRISPR/Cas9 cut. Red, sgRNA sequence. Clip images of sequence from Snap Gene viewer. **h**-**k**, Pictures of 24hpf WT (**h**), *CAMSAP2a*^{MO}; *CAMSAP3a*^{MO} (**i**), *CAMSAP2a*$^{-/-}$; *CAMSAP3a*$^{-/-}$ (**j**) and *CAMSAP2a*$^{-/-}$; *CAMSAP3a*^{MO} (**k**) zebrafish embryos. **l**, Western blot showing inputs and immunoprecipitations of GFP-Sara (CRISPR Knock in) and mVenus (expressed by CryA, see methods) with anti-GFP in two 5dpf zebrafish embryos. Number 1, positive embryo, number 2 negative embryo. Protein ladder, BenchMark.

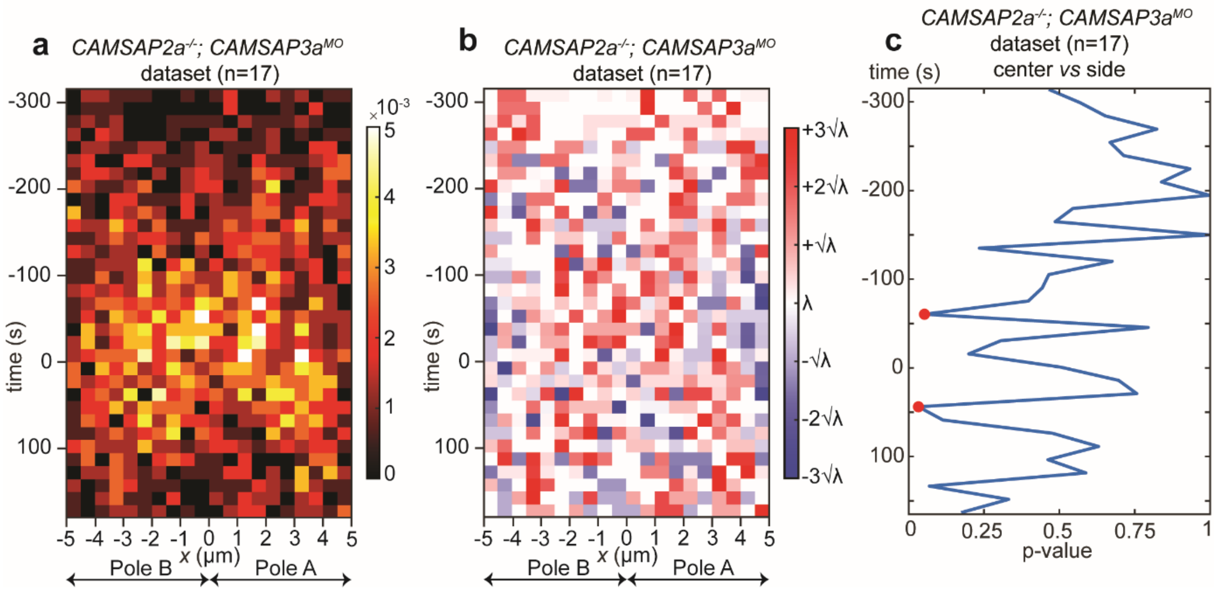

Figure S7: Heatmaps and ANOVA for CAMSAP2a$^{-/-}$; CAMSAP3a^{MO}dataset: **a**-**b**, Spatio-temporal density plot of Sara endosome binned number as a function of registered time for *CAMSAP2a*$^{-/-}$; *CAMSAP3a*^{MO} dataset as in Supplementary Fig. 3c,e-f (**a**) and Fig. 3b-c (**b**) NPs (n=17 NPs, 1523 endosomes). ANOVA comparison of Sara endosome mean densities as a function of registered time between cell center and cell sides for *CAMSAP2a*$^{-/-}$; *CAMSAP3a*^{MO} dataset as in Fig. 3d-f.

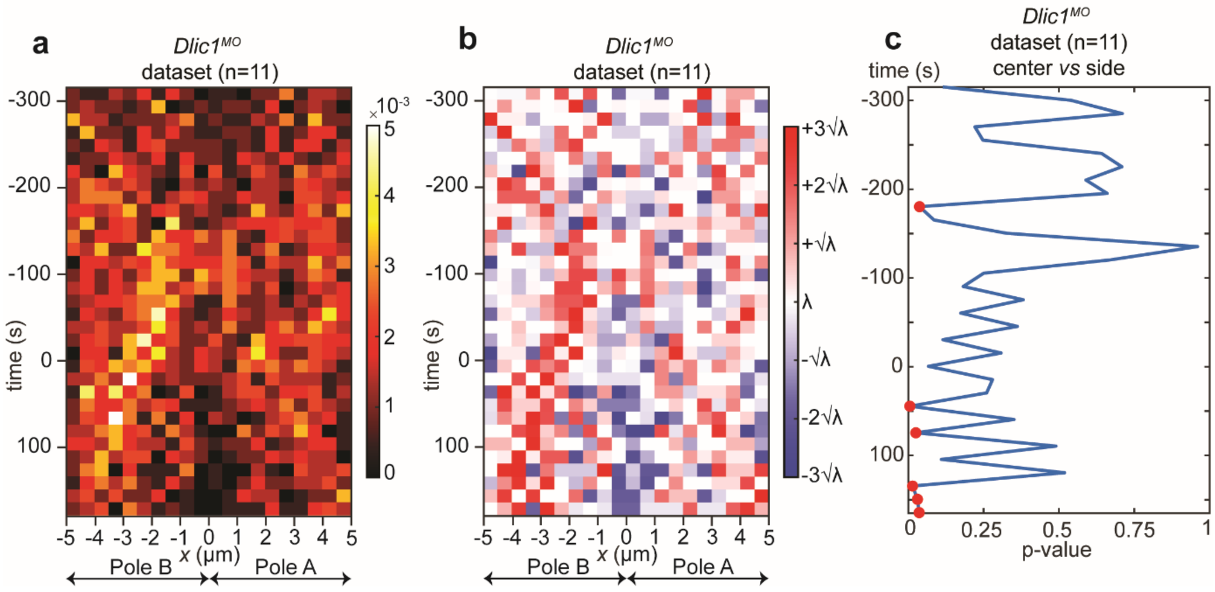

Figure S8: Heatmaps and ANOVA for Dlic1^{MO}dataset: **a**-**b**, Spatio-temporal density plot of Sara endosome binned number as a function of registered time for *Dlic*^{MO} dataset as in Supplementary Fig. 3c,e-f (**a**) and Fig. 3b-c (**b**) NPs (n=11 NPs, 2137 endosomes). **c**, ANOVA comparison of Sara endosome mean densities as a function of registered time between cell center and cell sides for *Dlic*^{MO} dataset as in Fig. 3d-f.

Supplementary Tables:

Supplementary Table 1: Zebrafish strains

Name	**Reference**
CAMSAP2a $^{-/-}$ *CAMSAP2b* $^{+/-}$	CRISPR KO
CAMSAP2a $^{-/-}$ *CAMSAP3a* $^{+/-}$	CRISPR KO
CAMSAP2a $^{+/-}$ *CAMSAP2b* $^{+/-}$	CRISPR KO
CAMSAP2a $^{+/-}$ *CAMSAP2b* $^{+/-}$	CRISPR KO
CAMSAP2a $^{+/-}$ *CAMSAP3a* $^{+/-}$	CRISPR KO
CAMSAP2a $^{-/-}$	CRISPR KO
CAMSAP3a $^{-/-}$	CRISPR KO
GFP:Sara Knock in	CRISPR KI
WT AB	ZDB-GENO-960809-7
βActin:*eGFP-DCX*	ZDB-ALT-170113-1

Supplementary Table 2: List of plasmids.

Plasmid name	Reference
pCS2 mCherry-CAMSAP2a	Made in lab
pCS2 mScarlet-CAMSAP3a	Made in lab
pCS2 N-ter Scarlet	Made in lab
pCS2 eGFP-DCX	Made in lab
pCS2 mCherry-Sara	Made in lab
pCS2 Par3-mCherry	Made in lab
pCS2 pSMOrange	Made in lab [1]
pCS2 C-ter mCherry	AddGene_34935 [2]
pCS2 N-ter eGFP	AddGene_34953 [2]
pCS2 N-ter mCherry	AddGene_34936 [2]
pCS2 mCherry-MKLP1	AddGene_140562 [3]
pCS2 KIF16Ba-mCherry	Made in lab
pCS2 KIF16Ba	Made in lab

Supplementary Table 3: List of primers.

Primer name	Seq. Ref.	Up/Low	Sequence
Fwd CAMPSAP2a cDNAamp	ZDB-GENE-030131-3016	up	GATCGGCGCGCCCCCATGGGAGATCTGAGCGAGTCCAGAGAC
Bwd CAMPSAP2a cDNAamp	ZDB-GENE-030131-3016	low	GATCGGCCGGCCCCCCTAGGACTTGACAGCCGCTACTTTCTT
Fwd CAMPSAP3a cDNAamp	ZDB-GENE-060503-811	up	GATCGGCGCGCCGATGGTGGACTCCAATGCAATGAGGAAG
Bwd CAMPSAP3a cDNAamp	ZDB-GENE-060503-811	low	GATCGGCCGGCCTTATTTTGGGGTCCCGAGCTTCTTTGG
Fwd Pard3ab	ZDB-GENE-030925-47	up	GATCGGCGCGCCCATGAAAGTGACGGTGTGTTTTGGGAGAACT

Bwd Pard3ab	ZDB-GENE-030925-47	low	GATCGGCCGGCCTTTGTACCTGTCTGAAGTGGAGGGGTGTCC
Fwd KIF16Ba cDNAamp	ZDB-GENE-090313-233	up	GATCGGCGCGCCCATGGCATCGGTCCGGGTGGCA
Bwd KIF16Ba cDNAamp	ZDB-GENE-090313-233	low	GATCGGCCGGCCTGAGCCCGTCCCGTGGCTGCT
Fwd DCX	AddGene_32852[4]	up	GATCGGCGCGCCCCATGGAACTTGATTTTGGACAC
Bwd DCX	AddGene_32852[4]	low	GATCGGCCGGCCCTTACATGGAATCACCAAGCG
Fwd pSMOrange	AddGene_31920 [1]	up	GATCGGCGCGCCCGCCACCATGGTGAGCAAGGGCGAGGAG
Bwd pSMOrange	AddGene_31920 [1]	low	GATCGGCCGGCCGTTACTTGTACAGCTCGTCCATGCCGCC
Fwd N-ter Scarlet	AddGene_85042 [5]	up	GATCGGATCCGCCACCATGGTGAGCAAGGGC
Bwd N-ter Scarlet	AddGene_85042 [5]	low	GATCACTAGTCTTGTACAGCTCGTCCATGCC

Supplementary Table 4: List of Morpholinos.

Morpholino name	Sequence
Camsap2a MO	5’-CTCCCATATTCACAACATCCAGCGA-3’
Camsap3a MO	5’-CGCGTCCATCGCCATTACTCAAGAC-3’
KIF16Ba MO	5’-TGACTTCCCTGATCCAGTCTGAC-3’
Par3 MO [6]	5’- TCAAAGGCTCCCGTGCTCTGGTGTC-3’
Dlic1 MO [7]	5’-GTGTATTTCTGCCCGTCGTCGCCA-3’
5-mispair CAMSAP2a ctrl MO	5’-CTCCgATATTgACAAgATCgAcCGT-3’

5-mispair CAMSAP3a ctrl MO	5'-CGCcTgCATCcCCATTACTgAAcAC-3'

Supplementary Table 5: List of KO sgRNAs and primers for genotyping.

Gene / primers	Exon	Mutation	sgRNA	WT Ref
CAMPSAP2a	12	+7bp ins	GGAGAG CAAAACA CCAGTG	ZDB-GENE-030131-3016
CAMSAP2a Check primers	Up: AGGAGTAGATGGAGCGTCAC		Low: CTCTGAGCTCTCGTACCTGC	
CAMPSAP3a	11	-5bp del	CTCGCAT AACACAA CCTCG	ZDB-GENE-060503-811
CAMSAP3a Check primers	Up : CACTTGCTGAGTAAGGCCCC		Low : GTTGCCGGTGTTTGGCAAAG	

Supplementary Table 6: List of Sara KI sgRNA and primers for genotyping.

Name	Sequence
ZiFiT antisense positive sgRNA	GGAGAATTACTTCCAGGCTG
Up SaraKI checking primer intron2	ATCCTGCACTGAATGCACAC
Low SaraKI checking primer intron2	CATGCTCGAGGAGAATTGGTGTGTCCGTTTC
Up SaraKI checking primer intron3	CAGCTCGATGCGGTTCACCAG
Low SaraKI checking primer intron3	AAAGAGACCGAGCTTTACTG

Supplementary References:

1. Subach, O. M., Entenberg, D., Condeelis, J. S. & Verkhusha, V. V. A FRET-facilitated photoswitching using an orange fluorescent protein with the fast photoconversion kinetics. *J. Am. Chem. Soc.* **134**, 14789–14799 (2012).
2. Gökirmak, T. *et al.* Localization and substrate selectivity of sea urchin multidrug (MDR) efflux transporters. *J. Biol. Chem.* **287**, 43876–43883 (2012).
3. Rathbun, L. I. *et al.* Cytokinetic bridge triggers de novo lumen formation in vivo. *Nat. Commun.* **11**, 1269 (2020).
4. Tanaka, T. *et al.* Lis1 and doublecortin function with dynein to mediate coupling of the nucleus to the centrosome in neuronal migration. *J. Cell Biol.* **165**, 709–721 (2004).
5. Bindels, D. S. *et al.* MScarlet: A bright monomeric red fluorescent protein for cellular imaging. *Nat. Methods* **14**, 53–56 (2016).
6. Wei, X. *et al.* The zebrafish Pard3 ortholog is required for separation of the eye fields and retinal lamination. *Dev. Biol.* **269**, 286–301 (2004).
7. Zhao, X. *et al.* Polarized endosome dynamics engage cytoplasmic Par-3 that recruits dynein during asymmetric cell division. *Sci. Adv.* **7**, 1–15 (2021).
