# Supplementary material for: Microtubule polarity determines the lineage of embryonic neural precursor in zebrafish spinal cord
Source: Commun Biol. 2024 Apr 10;7:439. doi: 10.1038/s42003-024-06018-7 (PMC11006876; doi:10.1038/s42003-024-06018-7)
Supplement: Supplementary file 2 — Description of Additional Supplementary Files [file 42003_2024_6018_MOESM2_ESM.pdf]

Description of Additional Supplementary Files

File name: Supplementary Data 1

Description: The data represented in each figure of the file, all raw images used in the manuscript and the results of sequencing.
