# Supplementary figures and images for: Microtubule polarity determines the lineage of embryonic neural precursor in zebrafish spinal cord
Source: Commun Biol. 2024 Apr 10;7:439. doi: 10.1038/s42003-024-06018-7 (PMC11006876; doi:10.1038/s42003-024-06018-7)

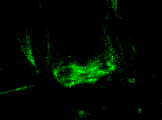

Supplement: Supplementary file 3 — Supplementary Data 1 [file 42003_2024_6018_MOESM3_ESM.zip › 22276_1_data_set_628581_s9vq7p/Supplementary Data 1/Microscope images/Figure1/1A/GFP-DCX/DCX 0.tif]

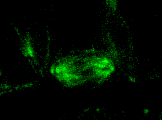

Supplement: Supplementary file 3 — Supplementary Data 1 [file 42003_2024_6018_MOESM3_ESM.zip › 22276_1_data_set_628581_s9vq7p/Supplementary Data 1/Microscope images/Figure1/1A/GFP-DCX/DCX -105.tif]

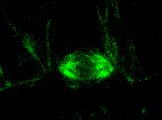

Supplement: Supplementary file 3 — Supplementary Data 1 [file 42003_2024_6018_MOESM3_ESM.zip › 22276_1_data_set_628581_s9vq7p/Supplementary Data 1/Microscope images/Figure1/1A/GFP-DCX/DCX -180.tif]

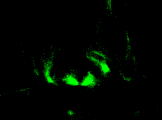

Supplement: Supplementary file 3 — Supplementary Data 1 [file 42003_2024_6018_MOESM3_ESM.zip › 22276_1_data_set_628581_s9vq7p/Supplementary Data 1/Microscope images/Figure1/1A/GFP-DCX/DCX 240.tif]

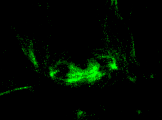

Supplement: Supplementary file 3 — Supplementary Data 1 [file 42003_2024_6018_MOESM3_ESM.zip › 22276_1_data_set_628581_s9vq7p/Supplementary Data 1/Microscope images/Figure1/1A/GFP-DCX/DCX 45.tif]

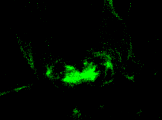

Supplement: Supplementary file 3 — Supplementary Data 1 [file 42003_2024_6018_MOESM3_ESM.zip › 22276_1_data_set_628581_s9vq7p/Supplementary Data 1/Microscope images/Figure1/1A/GFP-DCX/DCX 90.tif]

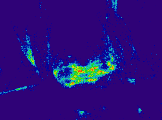

Supplement: Supplementary file 3 — Supplementary Data 1 [file 42003_2024_6018_MOESM3_ESM.zip › 22276_1_data_set_628581_s9vq7p/Supplementary Data 1/Microscope images/Figure1/1A/LUT/LUT 0.tif]

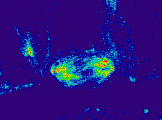

Supplement: Supplementary file 3 — Supplementary Data 1 [file 42003_2024_6018_MOESM3_ESM.zip › 22276_1_data_set_628581_s9vq7p/Supplementary Data 1/Microscope images/Figure1/1A/LUT/LUT -105.tif]

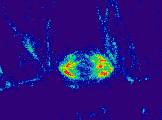

Supplement: Supplementary file 3 — Supplementary Data 1 [file 42003_2024_6018_MOESM3_ESM.zip › 22276_1_data_set_628581_s9vq7p/Supplementary Data 1/Microscope images/Figure1/1A/LUT/LUT -180.tif]

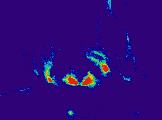

Supplement: Supplementary file 3 — Supplementary Data 1 [file 42003_2024_6018_MOESM3_ESM.zip › 22276_1_data_set_628581_s9vq7p/Supplementary Data 1/Microscope images/Figure1/1A/LUT/LUT 240.tif]

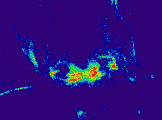

Supplement: Supplementary file 3 — Supplementary Data 1 [file 42003_2024_6018_MOESM3_ESM.zip › 22276_1_data_set_628581_s9vq7p/Supplementary Data 1/Microscope images/Figure1/1A/LUT/LUT 45.tif]

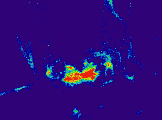

Supplement: Supplementary file 3 — Supplementary Data 1 [file 42003_2024_6018_MOESM3_ESM.zip › 22276_1_data_set_628581_s9vq7p/Supplementary Data 1/Microscope images/Figure1/1A/LUT/LUT 90.tif]

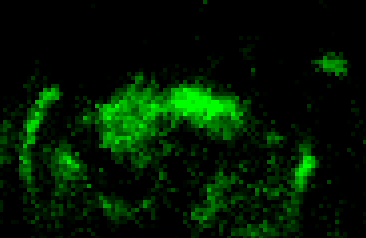

Supplement: Supplementary file 3 — Supplementary Data 1 [file 42003_2024_6018_MOESM3_ESM.zip › 22276_1_data_set_628581_s9vq7p/Supplementary Data 1/Microscope images/Figure1/1E/Asym cell/DCX.tif]

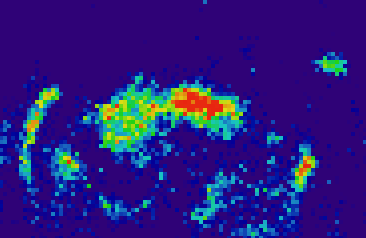

Supplement: Supplementary file 3 — Supplementary Data 1 [file 42003_2024_6018_MOESM3_ESM.zip › 22276_1_data_set_628581_s9vq7p/Supplementary Data 1/Microscope images/Figure1/1E/Asym cell/LUT.tif]

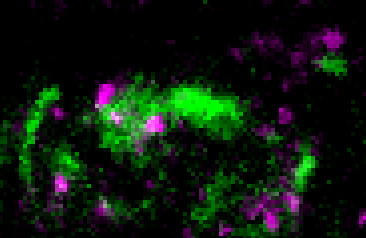

Supplement: Supplementary file 3 — Supplementary Data 1 [file 42003_2024_6018_MOESM3_ESM.zip › 22276_1_data_set_628581_s9vq7p/Supplementary Data 1/Microscope images/Figure1/1E/Asym cell/Merge.tif]

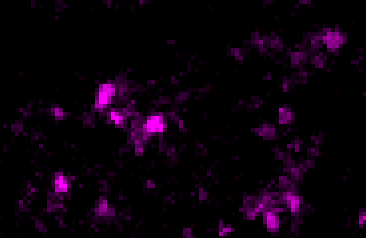

Supplement: Supplementary file 3 — Supplementary Data 1 [file 42003_2024_6018_MOESM3_ESM.zip › 22276_1_data_set_628581_s9vq7p/Supplementary Data 1/Microscope images/Figure1/1E/Asym cell/Sara.tif]

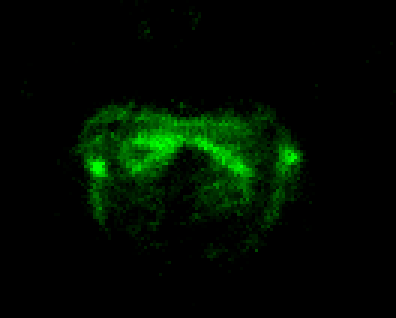

Supplement: Supplementary file 3 — Supplementary Data 1 [file 42003_2024_6018_MOESM3_ESM.zip › 22276_1_data_set_628581_s9vq7p/Supplementary Data 1/Microscope images/Figure1/1E/Sym cell/DCX.tif]

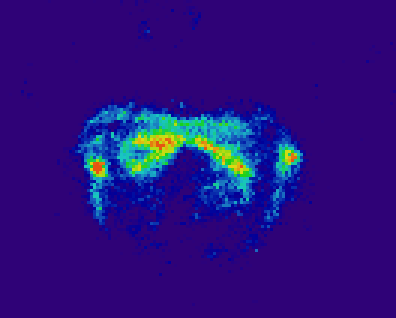

Supplement: Supplementary file 3 — Supplementary Data 1 [file 42003_2024_6018_MOESM3_ESM.zip › 22276_1_data_set_628581_s9vq7p/Supplementary Data 1/Microscope images/Figure1/1E/Sym cell/LUT.tif]

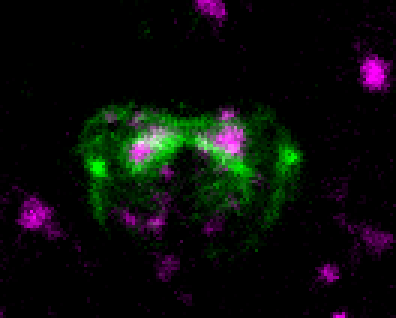

Supplement: Supplementary file 3 — Supplementary Data 1 [file 42003_2024_6018_MOESM3_ESM.zip › 22276_1_data_set_628581_s9vq7p/Supplementary Data 1/Microscope images/Figure1/1E/Sym cell/Merge.tif]

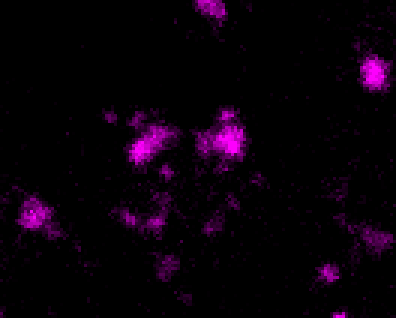

Supplement: Supplementary file 3 — Supplementary Data 1 [file 42003_2024_6018_MOESM3_ESM.zip › 22276_1_data_set_628581_s9vq7p/Supplementary Data 1/Microscope images/Figure1/1E/Sym cell/Sara.tif]

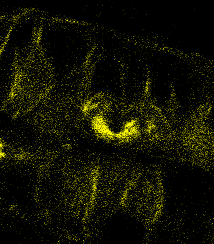

Supplement: Supplementary file 3 — Supplementary Data 1 [file 42003_2024_6018_MOESM3_ESM.zip › 22276_1_data_set_628581_s9vq7p/Supplementary Data 1/Microscope images/Figure2/2B/DCX.tif]

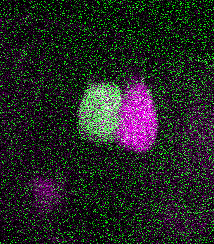

Supplement: Supplementary file 3 — Supplementary Data 1 [file 42003_2024_6018_MOESM3_ESM.zip › 22276_1_data_set_628581_s9vq7p/Supplementary Data 1/Microscope images/Figure2/2B/Merge.tif]

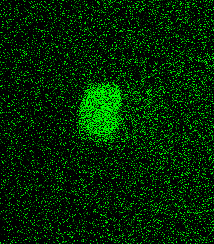

Supplement: Supplementary file 3 — Supplementary Data 1 [file 42003_2024_6018_MOESM3_ESM.zip › 22276_1_data_set_628581_s9vq7p/Supplementary Data 1/Microscope images/Figure2/2B/Photoconverted pSMOrange.tif]

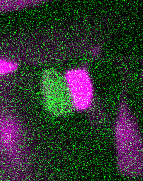

Supplement: Supplementary file 3 — Supplementary Data 1 [file 42003_2024_6018_MOESM3_ESM.zip › 22276_1_data_set_628581_s9vq7p/Supplementary Data 1/Microscope images/Figure2/2C/n.n/Merge.tif]

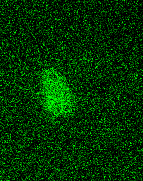

Supplement: Supplementary file 3 — Supplementary Data 1 [file 42003_2024_6018_MOESM3_ESM.zip › 22276_1_data_set_628581_s9vq7p/Supplementary Data 1/Microscope images/Figure2/2C/n.n/Photoconverted pSMOrange.tif]

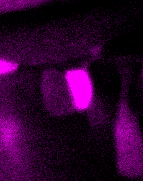

Supplement: Supplementary file 3 — Supplementary Data 1 [file 42003_2024_6018_MOESM3_ESM.zip › 22276_1_data_set_628581_s9vq7p/Supplementary Data 1/Microscope images/Figure2/2C/n.n/pSMOrange.tif]

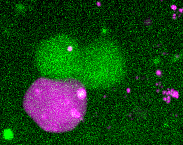

Supplement: Supplementary file 3 — Supplementary Data 1 [file 42003_2024_6018_MOESM3_ESM.zip › 22276_1_data_set_628581_s9vq7p/Supplementary Data 1/Microscope images/Figure2/2C/n.p/Merge.tif]

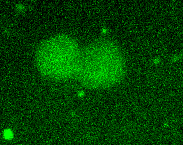

Supplement: Supplementary file 3 — Supplementary Data 1 [file 42003_2024_6018_MOESM3_ESM.zip › 22276_1_data_set_628581_s9vq7p/Supplementary Data 1/Microscope images/Figure2/2C/n.p/Photoconverted pSMOrange.tif]

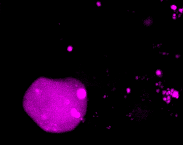

Supplement: Supplementary file 3 — Supplementary Data 1 [file 42003_2024_6018_MOESM3_ESM.zip › 22276_1_data_set_628581_s9vq7p/Supplementary Data 1/Microscope images/Figure2/2C/n.p/pSMOrange.tif]

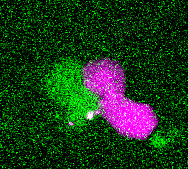

Supplement: Supplementary file 3 — Supplementary Data 1 [file 42003_2024_6018_MOESM3_ESM.zip › 22276_1_data_set_628581_s9vq7p/Supplementary Data 1/Microscope images/Figure2/2C/p.p/Merge.tif]

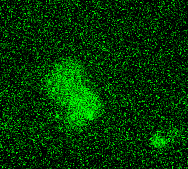

Supplement: Supplementary file 3 — Supplementary Data 1 [file 42003_2024_6018_MOESM3_ESM.zip › 22276_1_data_set_628581_s9vq7p/Supplementary Data 1/Microscope images/Figure2/2C/p.p/Photoconverted pSMOrange.tif]

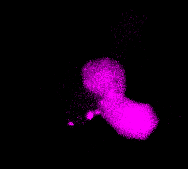

Supplement: Supplementary file 3 — Supplementary Data 1 [file 42003_2024_6018_MOESM3_ESM.zip › 22276_1_data_set_628581_s9vq7p/Supplementary Data 1/Microscope images/Figure2/2C/p.p/pSMOrange.tif]

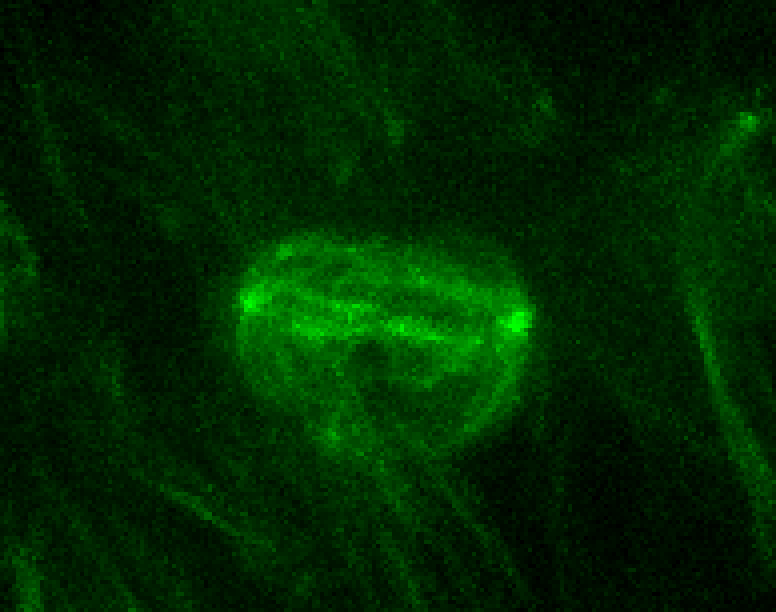

Supplement: Supplementary file 3 — Supplementary Data 1 [file 42003_2024_6018_MOESM3_ESM.zip › 22276_1_data_set_628581_s9vq7p/Supplementary Data 1/Microscope images/Figure3/DCX/DCX 0.tif]

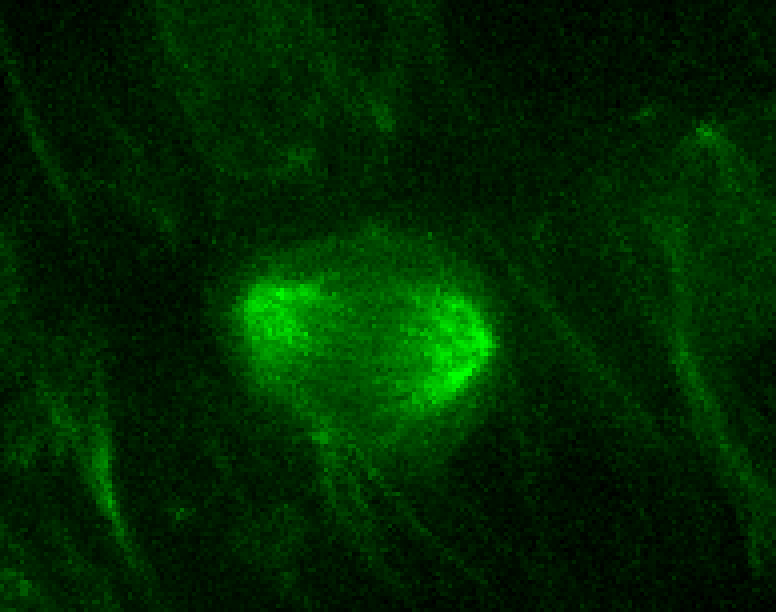

Supplement: Supplementary file 3 — Supplementary Data 1 [file 42003_2024_6018_MOESM3_ESM.zip › 22276_1_data_set_628581_s9vq7p/Supplementary Data 1/Microscope images/Figure3/DCX/DCX -150.tif]

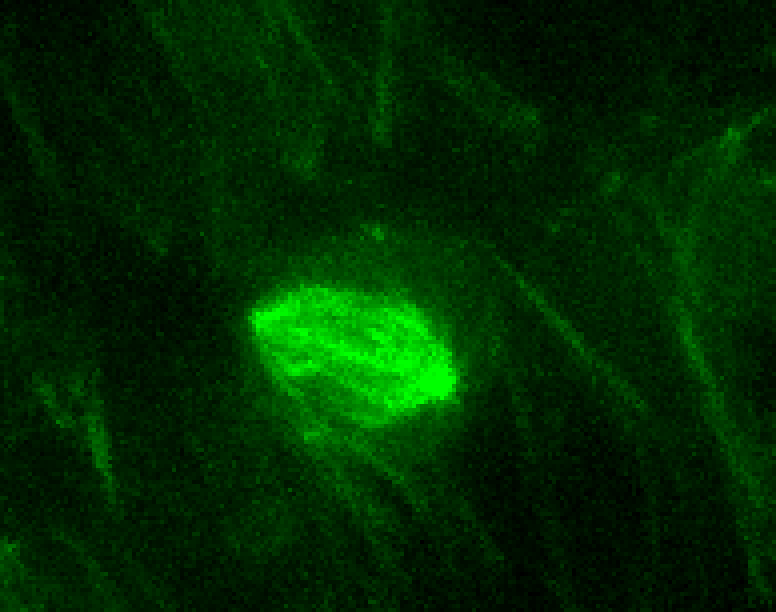

Supplement: Supplementary file 3 — Supplementary Data 1 [file 42003_2024_6018_MOESM3_ESM.zip › 22276_1_data_set_628581_s9vq7p/Supplementary Data 1/Microscope images/Figure3/DCX/DCX -300.tif]

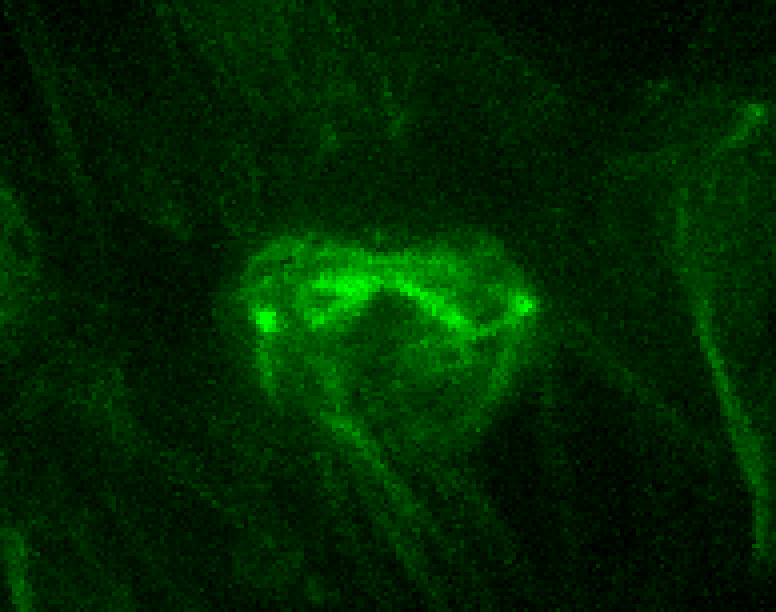

Supplement: Supplementary file 3 — Supplementary Data 1 [file 42003_2024_6018_MOESM3_ESM.zip › 22276_1_data_set_628581_s9vq7p/Supplementary Data 1/Microscope images/Figure3/DCX/DCX 75.tif]

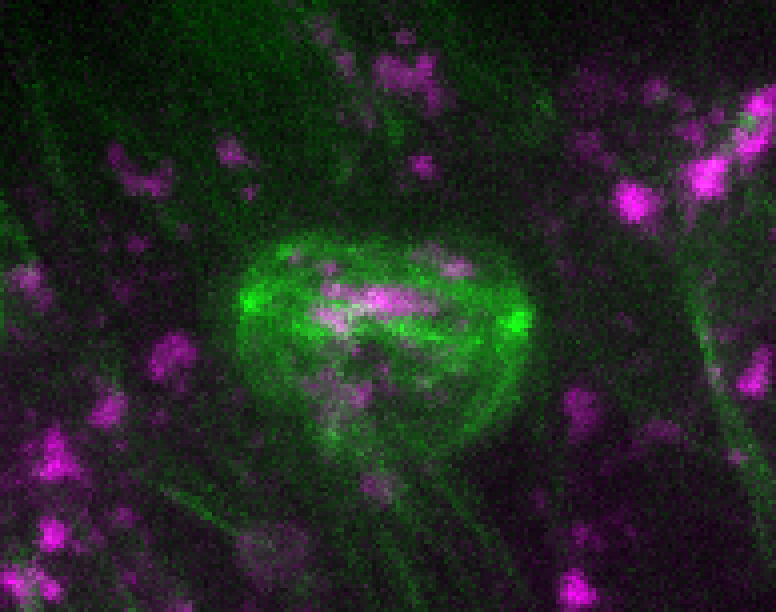

Supplement: Supplementary file 3 — Supplementary Data 1 [file 42003_2024_6018_MOESM3_ESM.zip › 22276_1_data_set_628581_s9vq7p/Supplementary Data 1/Microscope images/Figure3/Merge/Merge 0.tif]

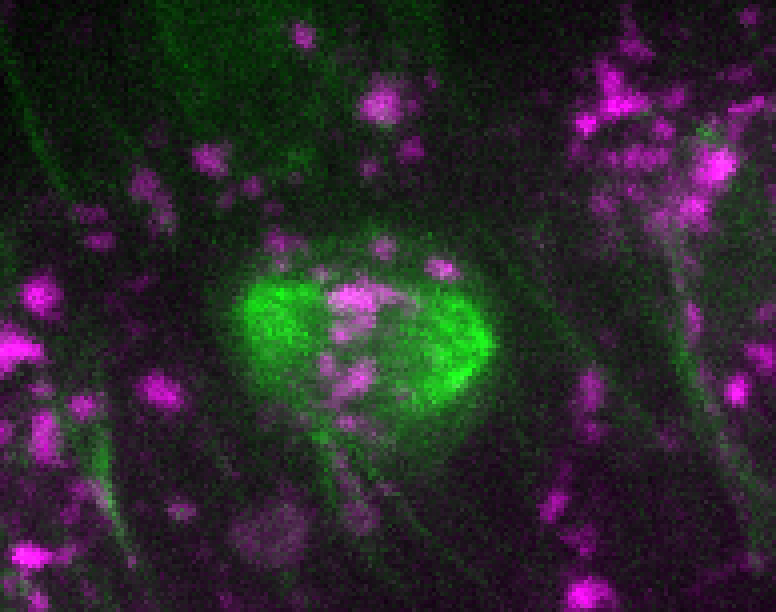

Supplement: Supplementary file 3 — Supplementary Data 1 [file 42003_2024_6018_MOESM3_ESM.zip › 22276_1_data_set_628581_s9vq7p/Supplementary Data 1/Microscope images/Figure3/Merge/Merge -150.tif]

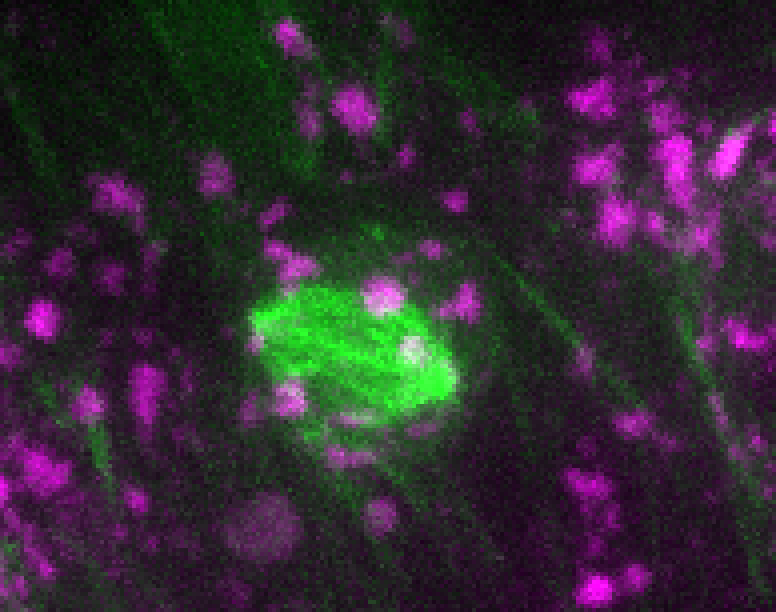

Supplement: Supplementary file 3 — Supplementary Data 1 [file 42003_2024_6018_MOESM3_ESM.zip › 22276_1_data_set_628581_s9vq7p/Supplementary Data 1/Microscope images/Figure3/Merge/Merge -300.tif]

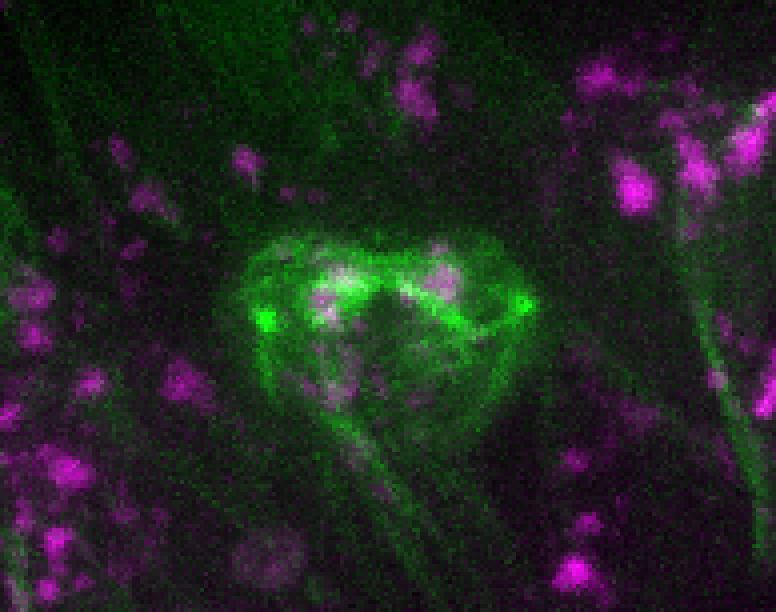

Supplement: Supplementary file 3 — Supplementary Data 1 [file 42003_2024_6018_MOESM3_ESM.zip › 22276_1_data_set_628581_s9vq7p/Supplementary Data 1/Microscope images/Figure3/Merge/Merge 75.tif]

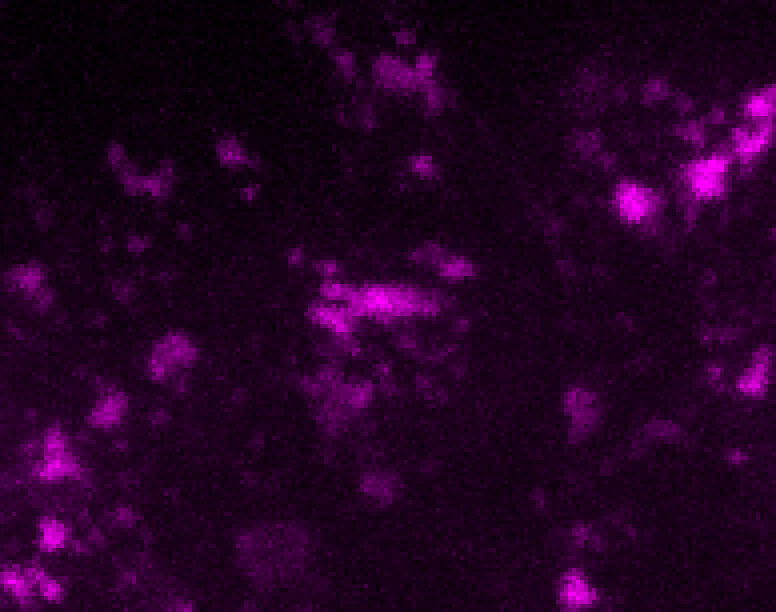

Supplement: Supplementary file 3 — Supplementary Data 1 [file 42003_2024_6018_MOESM3_ESM.zip › 22276_1_data_set_628581_s9vq7p/Supplementary Data 1/Microscope images/Figure3/Sara/Sara 0.tif]

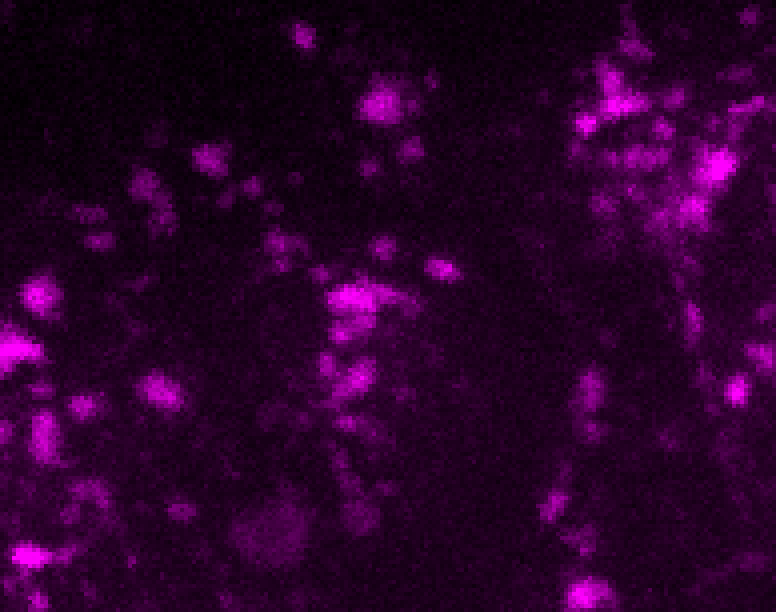

Supplement: Supplementary file 3 — Supplementary Data 1 [file 42003_2024_6018_MOESM3_ESM.zip › 22276_1_data_set_628581_s9vq7p/Supplementary Data 1/Microscope images/Figure3/Sara/Sara -150.tif]

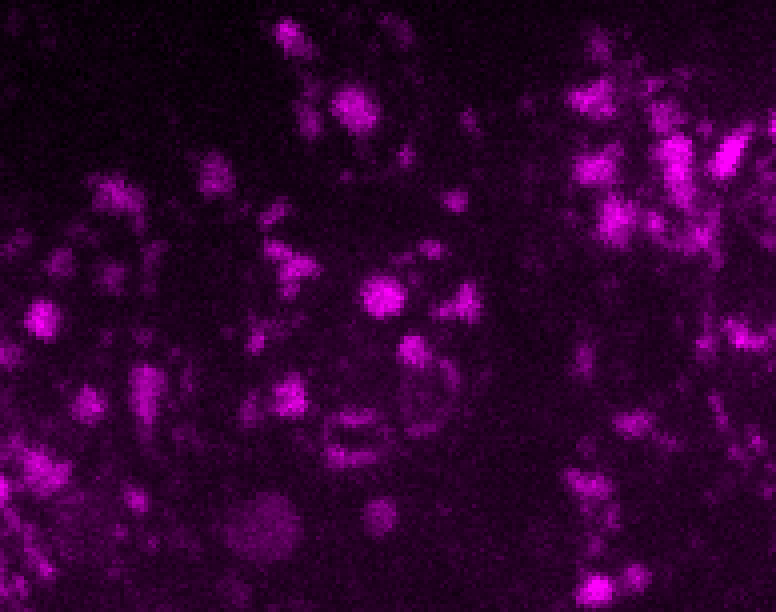

Supplement: Supplementary file 3 — Supplementary Data 1 [file 42003_2024_6018_MOESM3_ESM.zip › 22276_1_data_set_628581_s9vq7p/Supplementary Data 1/Microscope images/Figure3/Sara/Sara -300.tif]

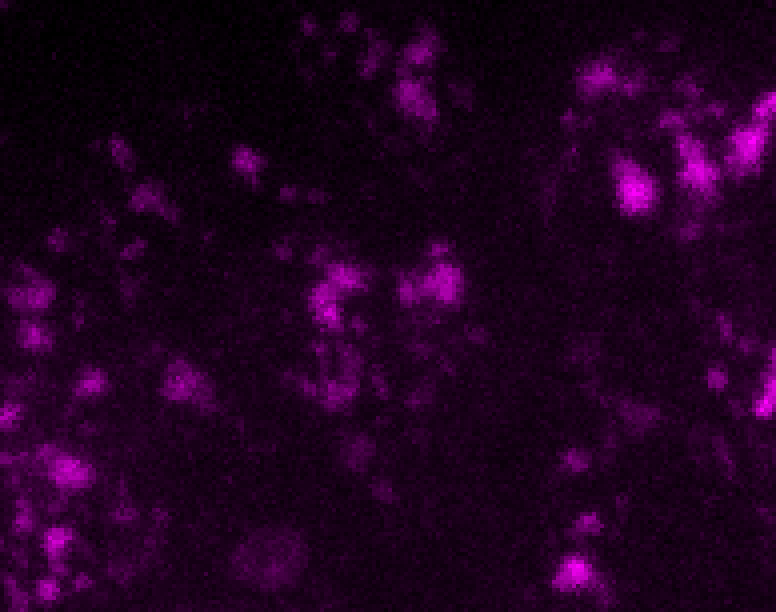

Supplement: Supplementary file 3 — Supplementary Data 1 [file 42003_2024_6018_MOESM3_ESM.zip › 22276_1_data_set_628581_s9vq7p/Supplementary Data 1/Microscope images/Figure3/Sara/Sara 75.tif]

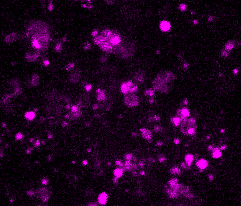

Supplement: Supplementary file 3 — Supplementary Data 1 [file 42003_2024_6018_MOESM3_ESM.zip › 22276_1_data_set_628581_s9vq7p/Supplementary Data 1/Microscope images/Figure4/4A/KIF16Ba.tif]

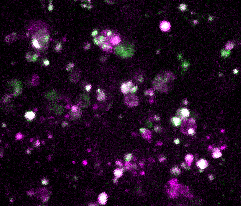

Supplement: Supplementary file 3 — Supplementary Data 1 [file 42003_2024_6018_MOESM3_ESM.zip › 22276_1_data_set_628581_s9vq7p/Supplementary Data 1/Microscope images/Figure4/4A/Merge.tif]

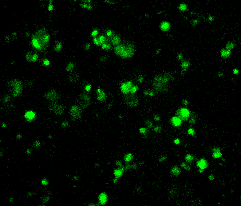

Supplement: Supplementary file 3 — Supplementary Data 1 [file 42003_2024_6018_MOESM3_ESM.zip › 22276_1_data_set_628581_s9vq7p/Supplementary Data 1/Microscope images/Figure4/4A/Sara.tif]

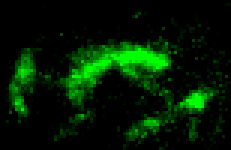

Supplement: Supplementary file 3 — Supplementary Data 1 [file 42003_2024_6018_MOESM3_ESM.zip › 22276_1_data_set_628581_s9vq7p/Supplementary Data 1/Microscope images/Figure4/4C/DCX.tif]

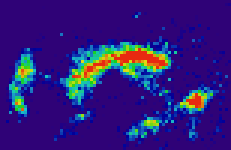

Supplement: Supplementary file 3 — Supplementary Data 1 [file 42003_2024_6018_MOESM3_ESM.zip › 22276_1_data_set_628581_s9vq7p/Supplementary Data 1/Microscope images/Figure4/4C/LUT.tif]

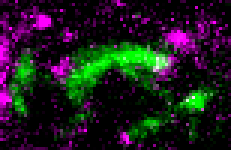

Supplement: Supplementary file 3 — Supplementary Data 1 [file 42003_2024_6018_MOESM3_ESM.zip › 22276_1_data_set_628581_s9vq7p/Supplementary Data 1/Microscope images/Figure4/4C/Merge.tif]

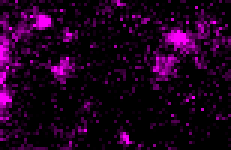

Supplement: Supplementary file 3 — Supplementary Data 1 [file 42003_2024_6018_MOESM3_ESM.zip › 22276_1_data_set_628581_s9vq7p/Supplementary Data 1/Microscope images/Figure4/4C/Sara.tif]

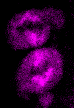

Supplement: Supplementary file 3 — Supplementary Data 1 [file 42003_2024_6018_MOESM3_ESM.zip › 22276_1_data_set_628581_s9vq7p/Supplementary Data 1/Microscope images/Figure5/5A/CAMSAP2a.tif]

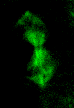

Supplement: Supplementary file 3 — Supplementary Data 1 [file 42003_2024_6018_MOESM3_ESM.zip › 22276_1_data_set_628581_s9vq7p/Supplementary Data 1/Microscope images/Figure5/5A/DCX.tif]

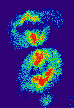

Supplement: Supplementary file 3 — Supplementary Data 1 [file 42003_2024_6018_MOESM3_ESM.zip › 22276_1_data_set_628581_s9vq7p/Supplementary Data 1/Microscope images/Figure5/5A/LUT CAMSAP2a.tif]

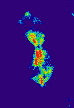

Supplement: Supplementary file 3 — Supplementary Data 1 [file 42003_2024_6018_MOESM3_ESM.zip › 22276_1_data_set_628581_s9vq7p/Supplementary Data 1/Microscope images/Figure5/5A/LUT DCX.tif]

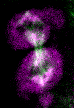

Supplement: Supplementary file 3 — Supplementary Data 1 [file 42003_2024_6018_MOESM3_ESM.zip › 22276_1_data_set_628581_s9vq7p/Supplementary Data 1/Microscope images/Figure5/5A/Merge.tif]

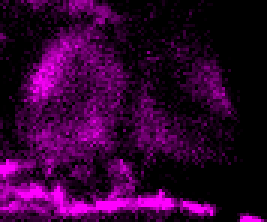

Supplement: Supplementary file 3 — Supplementary Data 1 [file 42003_2024_6018_MOESM3_ESM.zip › 22276_1_data_set_628581_s9vq7p/Supplementary Data 1/Microscope images/Figure5/5C/CAMSAP3a.tif]

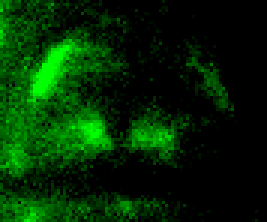

Supplement: Supplementary file 3 — Supplementary Data 1 [file 42003_2024_6018_MOESM3_ESM.zip › 22276_1_data_set_628581_s9vq7p/Supplementary Data 1/Microscope images/Figure5/5C/DCX.tif]

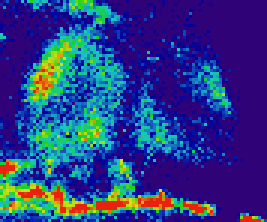

Supplement: Supplementary file 3 — Supplementary Data 1 [file 42003_2024_6018_MOESM3_ESM.zip › 22276_1_data_set_628581_s9vq7p/Supplementary Data 1/Microscope images/Figure5/5C/LUT CAMSAP3a.tif]

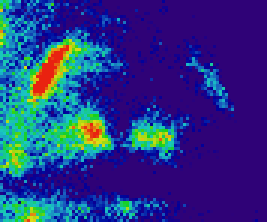

Supplement: Supplementary file 3 — Supplementary Data 1 [file 42003_2024_6018_MOESM3_ESM.zip › 22276_1_data_set_628581_s9vq7p/Supplementary Data 1/Microscope images/Figure5/5C/LUT DCX.tif]

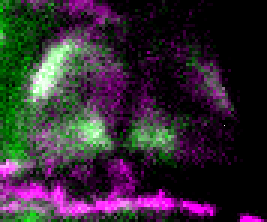

Supplement: Supplementary file 3 — Supplementary Data 1 [file 42003_2024_6018_MOESM3_ESM.zip › 22276_1_data_set_628581_s9vq7p/Supplementary Data 1/Microscope images/Figure5/5C/Merge.tif]

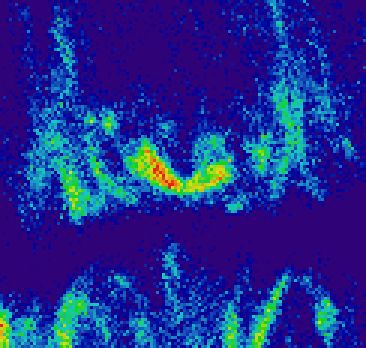

Supplement: Supplementary file 3 — Supplementary Data 1 [file 42003_2024_6018_MOESM3_ESM.zip › 22276_1_data_set_628581_s9vq7p/Supplementary Data 1/Microscope images/SupplementaryFigure1/S1E/DCX LUT.tif]

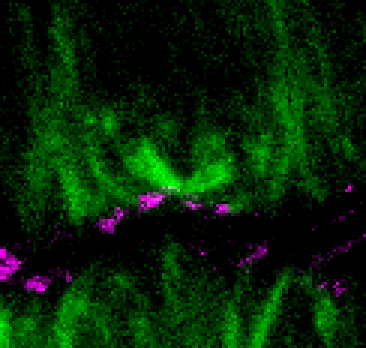

Supplement: Supplementary file 3 — Supplementary Data 1 [file 42003_2024_6018_MOESM3_ESM.zip › 22276_1_data_set_628581_s9vq7p/Supplementary Data 1/Microscope images/SupplementaryFigure1/S1E/Merge DCX and Par3.tif]

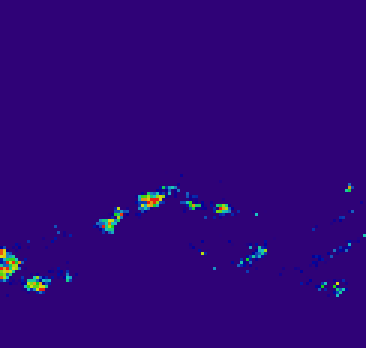

Supplement: Supplementary file 3 — Supplementary Data 1 [file 42003_2024_6018_MOESM3_ESM.zip › 22276_1_data_set_628581_s9vq7p/Supplementary Data 1/Microscope images/SupplementaryFigure1/S1E/Par3 LUT.tif]

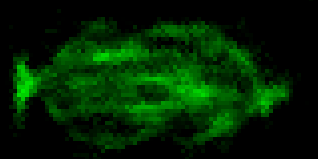

Supplement: Supplementary file 3 — Supplementary Data 1 [file 42003_2024_6018_MOESM3_ESM.zip › 22276_1_data_set_628581_s9vq7p/Supplementary Data 1/Microscope images/SupplementaryFigure3/S3G/DCX/DCX 0.tif]

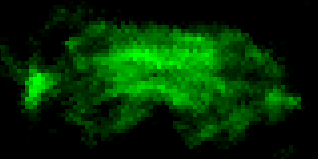

Supplement: Supplementary file 3 — Supplementary Data 1 [file 42003_2024_6018_MOESM3_ESM.zip › 22276_1_data_set_628581_s9vq7p/Supplementary Data 1/Microscope images/SupplementaryFigure3/S3G/DCX/DCX 15.tif]

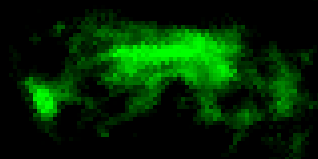

Supplement: Supplementary file 3 — Supplementary Data 1 [file 42003_2024_6018_MOESM3_ESM.zip › 22276_1_data_set_628581_s9vq7p/Supplementary Data 1/Microscope images/SupplementaryFigure3/S3G/DCX/DCX 30.tif]

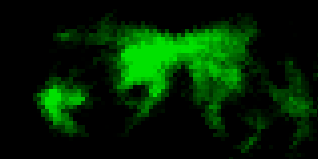

Supplement: Supplementary file 3 — Supplementary Data 1 [file 42003_2024_6018_MOESM3_ESM.zip › 22276_1_data_set_628581_s9vq7p/Supplementary Data 1/Microscope images/SupplementaryFigure3/S3G/DCX/DCX 45.tif]

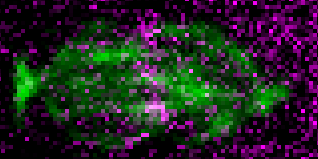

Supplement: Supplementary file 3 — Supplementary Data 1 [file 42003_2024_6018_MOESM3_ESM.zip › 22276_1_data_set_628581_s9vq7p/Supplementary Data 1/Microscope images/SupplementaryFigure3/S3G/Merge/Merge 0.tif]

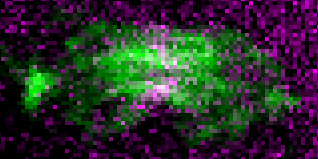

Supplement: Supplementary file 3 — Supplementary Data 1 [file 42003_2024_6018_MOESM3_ESM.zip › 22276_1_data_set_628581_s9vq7p/Supplementary Data 1/Microscope images/SupplementaryFigure3/S3G/Merge/Merge 15.tif]

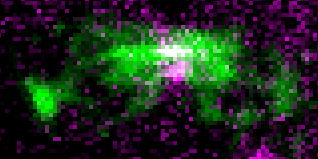

Supplement: Supplementary file 3 — Supplementary Data 1 [file 42003_2024_6018_MOESM3_ESM.zip › 22276_1_data_set_628581_s9vq7p/Supplementary Data 1/Microscope images/SupplementaryFigure3/S3G/Merge/Merge 30.tif]

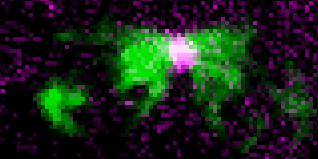

Supplement: Supplementary file 3 — Supplementary Data 1 [file 42003_2024_6018_MOESM3_ESM.zip › 22276_1_data_set_628581_s9vq7p/Supplementary Data 1/Microscope images/SupplementaryFigure3/S3G/Merge/Merge 45.tif]

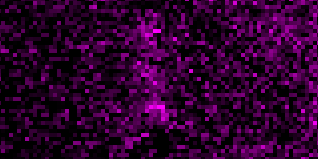

Supplement: Supplementary file 3 — Supplementary Data 1 [file 42003_2024_6018_MOESM3_ESM.zip › 22276_1_data_set_628581_s9vq7p/Supplementary Data 1/Microscope images/SupplementaryFigure3/S3G/MKLP1/MKLP1 0.tif]

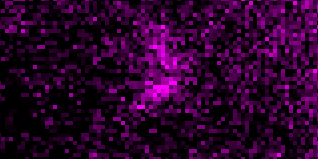

Supplement: Supplementary file 3 — Supplementary Data 1 [file 42003_2024_6018_MOESM3_ESM.zip › 22276_1_data_set_628581_s9vq7p/Supplementary Data 1/Microscope images/SupplementaryFigure3/S3G/MKLP1/MKLP1 15.tif]

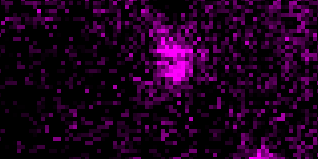

Supplement: Supplementary file 3 — Supplementary Data 1 [file 42003_2024_6018_MOESM3_ESM.zip › 22276_1_data_set_628581_s9vq7p/Supplementary Data 1/Microscope images/SupplementaryFigure3/S3G/MKLP1/MKLP1 30.tif]

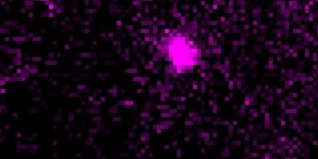

Supplement: Supplementary file 3 — Supplementary Data 1 [file 42003_2024_6018_MOESM3_ESM.zip › 22276_1_data_set_628581_s9vq7p/Supplementary Data 1/Microscope images/SupplementaryFigure3/S3G/MKLP1/MKLP1 45.tif]

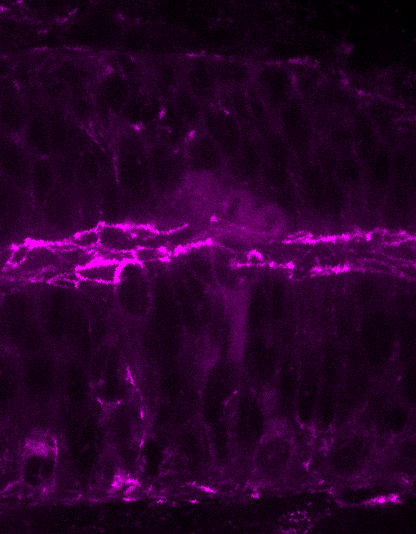

Supplement: Supplementary file 3 — Supplementary Data 1 [file 42003_2024_6018_MOESM3_ESM.zip › 22276_1_data_set_628581_s9vq7p/Supplementary Data 1/Microscope images/SupplementaryFigure6/S6B/CAMSAP3a.tif]

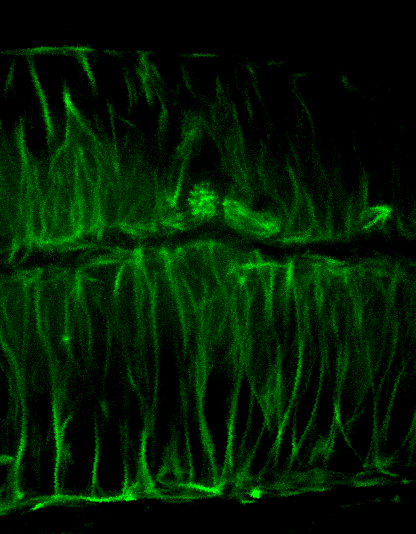

Supplement: Supplementary file 3 — Supplementary Data 1 [file 42003_2024_6018_MOESM3_ESM.zip › 22276_1_data_set_628581_s9vq7p/Supplementary Data 1/Microscope images/SupplementaryFigure6/S6B/DCX.tif]

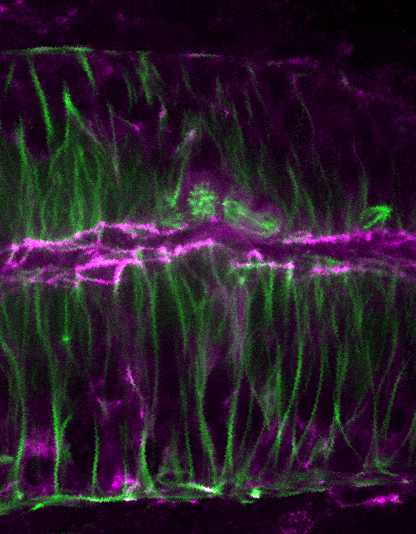

Supplement: Supplementary file 3 — Supplementary Data 1 [file 42003_2024_6018_MOESM3_ESM.zip › 22276_1_data_set_628581_s9vq7p/Supplementary Data 1/Microscope images/SupplementaryFigure6/S6B/Merge.tif]

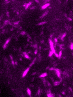

Supplement: Supplementary file 3 — Supplementary Data 1 [file 42003_2024_6018_MOESM3_ESM.zip › 22276_1_data_set_628581_s9vq7p/Supplementary Data 1/Microscope images/SupplementaryFigure6/S6C/CAMSAP3a.tif]

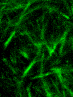

Supplement: Supplementary file 3 — Supplementary Data 1 [file 42003_2024_6018_MOESM3_ESM.zip › 22276_1_data_set_628581_s9vq7p/Supplementary Data 1/Microscope images/SupplementaryFigure6/S6C/DCX.tif]

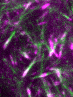

Supplement: Supplementary file 3 — Supplementary Data 1 [file 42003_2024_6018_MOESM3_ESM.zip › 22276_1_data_set_628581_s9vq7p/Supplementary Data 1/Microscope images/SupplementaryFigure6/S6C/Merge.tif]

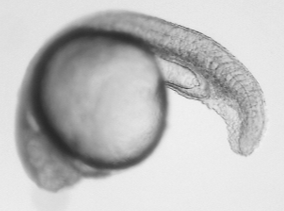

Supplement: Supplementary file 3 — Supplementary Data 1 [file 42003_2024_6018_MOESM3_ESM.zip › 22276_1_data_set_628581_s9vq7p/Supplementary Data 1/Microscope images/SupplementaryFigure6/S6H-K/CAMSAP2aKO CAMSAP3aKOtif.tif]

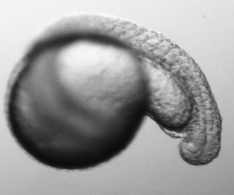

Supplement: Supplementary file 3 — Supplementary Data 1 [file 42003_2024_6018_MOESM3_ESM.zip › 22276_1_data_set_628581_s9vq7p/Supplementary Data 1/Microscope images/SupplementaryFigure6/S6H-K/CAMSAP2aKO CAMSAP3aMOtif.tif]

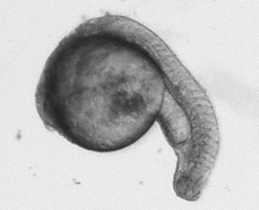

Supplement: Supplementary file 3 — Supplementary Data 1 [file 42003_2024_6018_MOESM3_ESM.zip › 22276_1_data_set_628581_s9vq7p/Supplementary Data 1/Microscope images/SupplementaryFigure6/S6H-K/CAMSAP2aMO CAMSAP3aMOtif.tif]

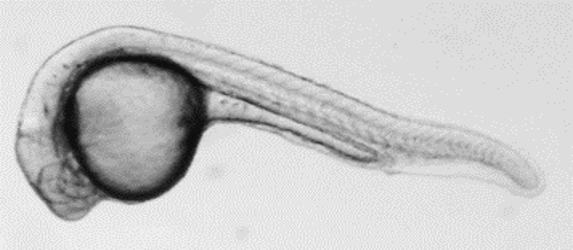

Supplement: Supplementary file 3 — Supplementary Data 1 [file 42003_2024_6018_MOESM3_ESM.zip › 22276_1_data_set_628581_s9vq7p/Supplementary Data 1/Microscope images/SupplementaryFigure6/S6H-K/WT.tif]

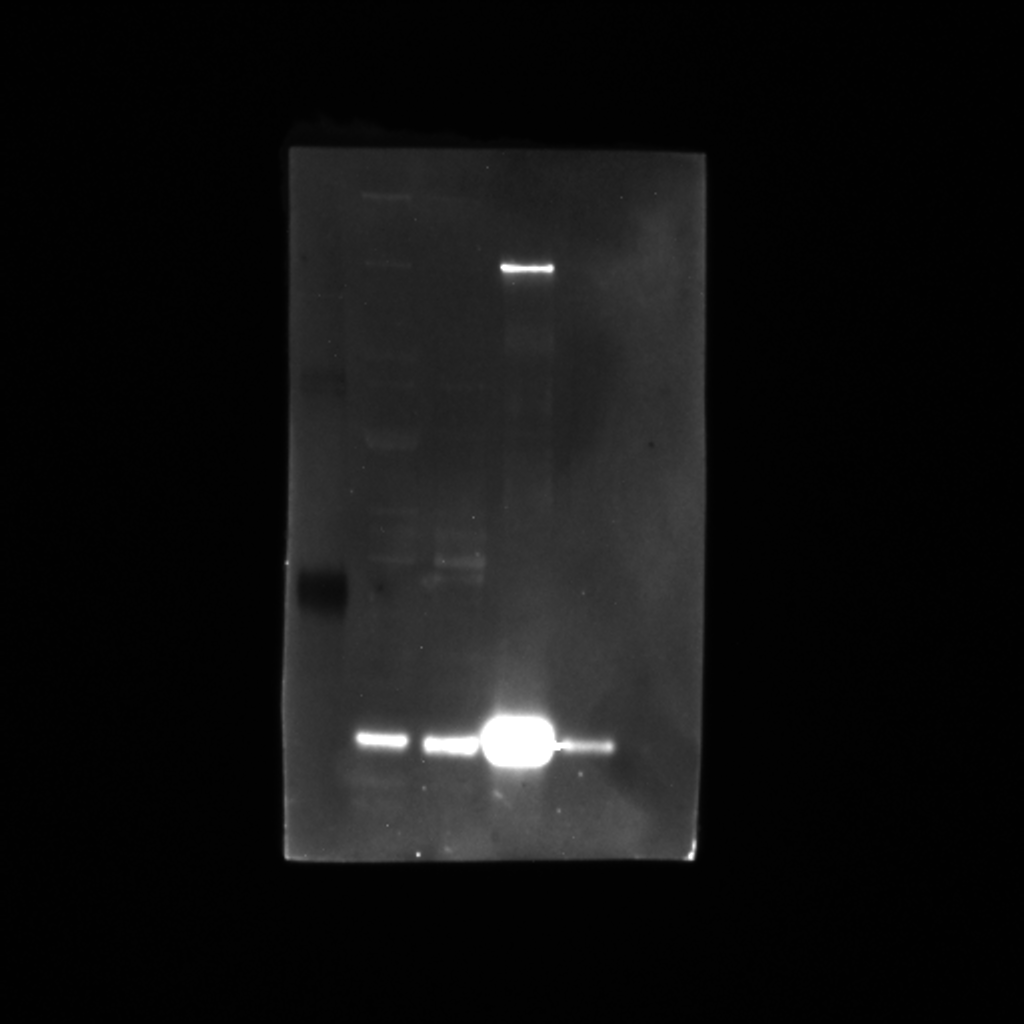

Supplement: Supplementary file 3 — Supplementary Data 1 [file 42003_2024_6018_MOESM3_ESM.zip › 22276_1_data_set_628581_s9vq7p/Supplementary Data 1/Microscope images/SupplementaryFigure6/S6I/5min anti GFP.Tif]

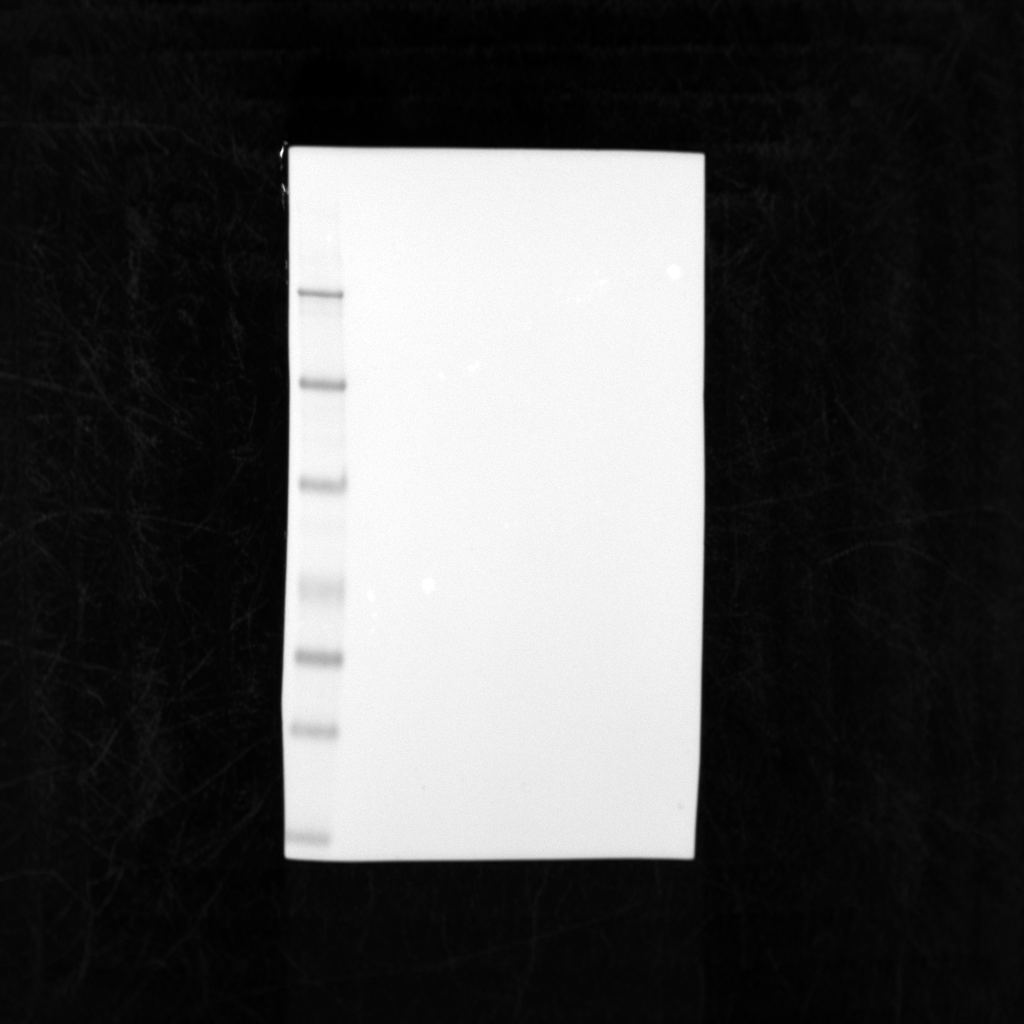

Supplement: Supplementary file 3 — Supplementary Data 1 [file 42003_2024_6018_MOESM3_ESM.zip › 22276_1_data_set_628581_s9vq7p/Supplementary Data 1/Microscope images/SupplementaryFigure6/S6I/Ladder.Tif]

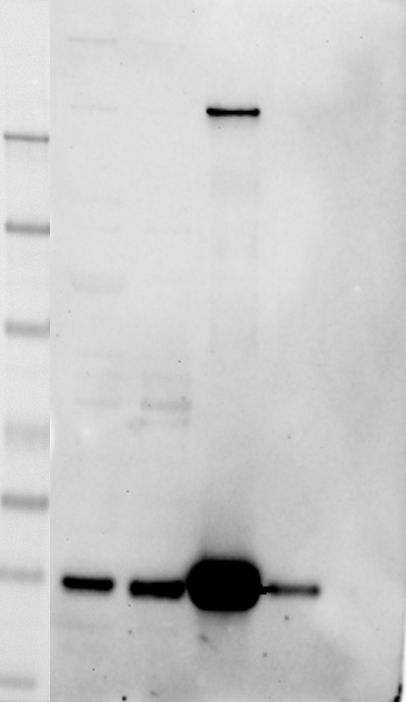

Supplement: Supplementary file 3 — Supplementary Data 1 [file 42003_2024_6018_MOESM3_ESM.zip › 22276_1_data_set_628581_s9vq7p/Supplementary Data 1/Microscope images/SupplementaryFigure6/S6I/Merge.tif]
