# Supplementary material for: Microtubule polarity determines the lineage of embryonic neural precursor in zebrafish spinal cord
Source: Commun Biol. 2024 Apr 10;7:439. doi: 10.1038/s42003-024-06018-7 (PMC11006876; doi:10.1038/s42003-024-06018-7)
Supplement: Supplementary file 4 — Reporting Summary [file 42003_2024_6018_MOESM4_ESM.pdf]

nature portfolio

Corresponding author(s): Marcos Gonzalez-Gaitan

Last updated by author(s): Feb 8, 2024

Reporting Summary

Nature Portfolio wishes to improve the reproducibility of the work that we publish. This form provides structure for consistency and transparency in reporting. For further information on Nature Portfolio policies, see our Editorial Policies and the Editorial Policy Checklist.

Statistics

For all statistical analyses, confirm that the following items are present in the figure legend, table legend, main text, or Methods section.

n/a	Confirmed	
☐	☒	The exact sample size (*n*) for each experimental group/condition, given as a discrete number and unit of measurement
☐	☒	A statement on whether measurements were taken from distinct samples or whether the same sample was measured repeatedly
☐	☒	The statistical test(s) used AND whether they are one- or two-sided<br>*Only common tests should be described solely by name; describe more complex techniques in the Methods section.*
☒	☐	A description of all covariates tested
☐	☒	A description of any assumptions or corrections, such as tests of normality and adjustment for multiple comparisons
☐	☒	A full description of the statistical parameters including central tendency (e.g. means) or other basic estimates (e.g. regression coefficient) AND variation (e.g. standard deviation) or associated estimates of uncertainty (e.g. confidence intervals)
☐	☒	For null hypothesis testing, the test statistic (e.g. *F*, *t*, *r*) with confidence intervals, effect sizes, degrees of freedom and *P* value noted<br>*Give P values as exact values whenever suitable.*
☒	☐	For Bayesian analysis, information on the choice of priors and Markov chain Monte Carlo settings
☒	☐	For hierarchical and complex designs, identification of the appropriate level for tests and full reporting of outcomes
☒	☐	Estimates of effect sizes (e.g. Cohen's *d*, Pearson's *r*), indicating how they were calculated

Our web collection on statistics for biologists contains articles on many of the points above.

Software and code

Policy information about availability of computer code

Data collection	Software built in the microscope for image acquisition: Slidebook 6 (3i)
Data analysis	FiJi (2.14), Excel MS Office 2012 and MATLAB 2021b (The MathWorks) software were used for analysis. Figures were generated using Illustrator (Adobe 2021).<br>MSD analyzer (https://ch.mathworks.com/matlabcentral/fileexchange/40692-mean-square-displacement-analysis-of-particles-trajectories) was used to compute MSD analysis. Shaddederrorbar package (https://ch.mathworks.com/matlabcentral/fileexchange/26311-raacampbell-shadederrorbar) was used to generate shade of standard error on figures. Trackmate plugin on Fiji was used to obtain the tracks and coordinates of Sara endosomes (https://imagej.net/plugins/trackmate/)<br>Matlab and ImageJ codes from Derivery et al., Nature, 2015 were used and modified to acquire physical parameters of Sara endosome motility. Those custom codes written in ImageJ and Matlab are available upon request.

For manuscripts utilizing custom algorithms or software that are central to the research but not yet described in published literature, software must be made available to editors and reviewers. We strongly encourage code deposition in a community repository (e.g. GitHub). See the Nature Portfolio guidelines for submitting code & software for further information.

Data

Policy information about availability of data

All manuscripts must include a data availability statement. This statement should provide the following information, where applicable:

- Accession codes, unique identifiers, or web links for publicly available datasets
- A description of any restrictions on data availability
- For clinical datasets or third party data, please ensure that the statement adheres to our policy

All data supporting the findings of this study are available within the paper, Supplementary Data 1 for Supplementary Figures and Supplementary Tables, and Supplementary Data 2 for data point values and raw images used for analysis. All other data are available upon request.

Research involving human participants, their data, or biological material

Policy information about studies with human participants or human data. See also policy information about sex, gender (identity/presentation), and sexual orientation and race, ethnicity and racism.

Reporting on sex and gender	*Use the terms sex (biological attribute) and gender (shaped by social and cultural circumstances) carefully in order to avoid confusing both terms. Indicate if findings apply to only one sex or gender; describe whether sex and gender were considered in study design; whether sex and/or gender was determined based on self-reporting or assigned and methods used. Provide in the source data disaggregated sex and gender data, where this information has been collected, and if consent has been obtained for sharing of individual-level data; provide overall numbers in this Reporting Summary. Please state if this information has not been collected. Report sex- and gender-based analyses where performed, justify reasons for lack of sex- and gender-based analysis.*
Reporting on race, ethnicity, or other socially relevant groupings	*Please specify the socially constructed or socially relevant categorization variable(s) used in your manuscript and explain why they were used. Please note that such variables should not be used as proxies for other socially constructed/relevant variables (for example, race or ethnicity should not be used as a proxy for socioeconomic status). Provide clear definitions of the relevant terms used, how they were provided (by the participants/respondents, the researchers, or third parties), and the method(s) used to classify people into the different categories (e.g. self-report, census or administrative data, social media data, etc.) Please provide details about how you controlled for confounding variables in your analyses.*
Population characteristics	*Describe the covariate-relevant population characteristics of the human research participants (e.g. age, genotypic information, past and current diagnosis and treatment categories). If you filled out the behavioural & social sciences study design questions and have nothing to add here, write "See above."*
Recruitment	*Describe how participants were recruited. Outline any potential self-selection bias or other biases that may be present and how these are likely to impact results.*
Ethics oversight	*Identify the organization(s) that approved the study protocol.*

Note that full information on the approval of the study protocol must also be provided in the manuscript.

Field-specific reporting

Please select the one below that is the best fit for your research. If you are not sure, read the appropriate sections before making your selection.

☒ Life sciences ☐ Behavioural & social sciences ☐ Ecological, evolutionary & environmental sciences

For a reference copy of the document with all sections, see nature.com/documents/nr-reporting-summary-flat.pdf

Life sciences study design

All studies must disclose on these points even when the disclosure is negative.

Sample size	Sample size were not pre-calculated to perform our experiments. Sample sizes were based on comparison between control and mutant/ morphant conditions where the statistical significances could be efficiently measured. Sample size were chosen based on other neural precursor zebrafish works to have similar numbers for statistical tests.
Data exclusions	Refinement of the data for Sara endosome tracking and the parameters used are indicated in the methodology of the manuscript. This refinement was applied to avoid false detection of endosomes (too small, bad signal to noise ratio, false tracks). Embryos showing a strong toxicity (malformation, decomposition) related to injections were not considered.
Replication	All experiments are representative from a set of at least 2 independent experimental rounds performed on different days and include data replicates. Except for the lineage experiments, where only 1 neural precursor per embryo was imaged, a maximum of 3 neural precursors per zebrafish embryo were imaged.
Randomization	Zebrafish from the same AB genetic background were used from different batch and generations, and experiments were carried out on randomly chosen zebrafish embryos of the appropriate genetic background and developmental stage. Our study does not explore the impact

	of different treatments on subjects, nor did it require sampling individuals that belong to different groups from large populations. As such randomization is not strictly relevant to our analysis.
Blinding	No blinded studies were performed. Blinding is not relevant to this study. The experiments require the investigators to group the data between control and testing condition to quantify differences.

Reporting for specific materials, systems and methods

We require information from authors about some types of materials, experimental systems and methods used in many studies. Here, indicate whether each material, system or method listed is relevant to your study. If you are not sure if a list item applies to your research, read the appropriate section before selecting a response.

Materials & experimental systems

n/a	Involved in the study
☐	☒ Antibodies
☒	☐ Eukaryotic cell lines
☒	☐ Palaeontology and archaeology
☐	☒ Animals and other organisms
☒	☐ Clinical data
☒	☐ Dual use research of concern
☒	☐ Plants

Methods

n/a	Involved in the study
☒	☐ ChIP-seq
☒	☐ Flow cytometry
☒	☐ MRI-based neuroimaging

Antibodies

Antibodies used	mouse anti GFP antibody (Roche, ref. 11814460001)
Validation	Validation from the manufacture: https://www.sigmaaldrich.com/deepweb/assets/sigmaaldrich/product/documents/270/763/11814460001.pdf

Animals and other research organisms

Policy information about studies involving animals; ARRIVE guidelines recommended for reporting animal research, and Sex and Gender in Research

Laboratory animals	Zebrafish embryos were used in this work. All strains generated (CRISPR) or ordered from EZRC (https://www.ezrc.kit.edu/) and their references are indicated in Supplementary Data 1. Adult zebrafish between 3 months and 18 months old were used to produce embryos.
Wild animals	This study did not involve wild animals.
Reporting on sex	Sex of zebrafish embryos were not considered in this work.
Field-collected samples	This study did not involve samples collected from the field.
Ethics oversight	Housing and ethic are and monitored regularly by the Swiss veterinary office (https://www.blv.admin.ch/blv/en/home/tiere/tierversuche.html)

Note that full information on the approval of the study protocol must also be provided in the manuscript.
